# Fabrication, Characterization, and Microbial Biodegradation of Transparent Nanodehydrated Bioplastic (NDB) Membranes Using Novel Casting, Dehydration, and Peeling Techniques

**DOI:** 10.3390/polym15153303

**Published:** 2023-08-04

**Authors:** Sherif S. Hindi, Mona Othman I. Albureikan

**Affiliations:** 1 Department of Agriculture, Faculty of Environmental Sciences, King Abdulaziz University (KAU), P.O. Box 80208, Jeddah 21589, Saudi Arabia; 2 Department of Biological Sciences, Faculty of Science, King Abdulaziz University (KAU), P.O. Box 80208, Jeddah 21589, Saudi Arabia; malboraikan@kau.edu.sa

**Keywords:** hydrophilic bioplastics, gum Arabic, polyvinyl alcohol, casting of poly-(methyl methacrylate), dehydration, phosphorus pentoxide, peeling, microbial biodegradation

## Abstract

NDBs were fabricated from gum Arabic (GA) and polyvinyl alcohol (PVA) in different ratios using novel techniques (casting, dehydration, and peeling). The GA/PVA blends were cast with a novel vibration-free horizontal flow (VFHF) technique, producing membranes free of air bubble defects with a homogenous texture, smooth surface, and constant thickness. The casting process was achieved on a self-electrostatic template (SET) made of poly-(methyl methacrylate), which made peeling the final product membranes easy due to its non-stick behavior. After settling the casting of the membranous, while blind, the sheets were dried using nanometric dehydration under a mild vacuum stream using a novel stratified nano-dehydrator (SND) loaded with P_2_O_5_. After drying the NDB, the dry, smooth membranes were peeled easily without scratching defects. The physicochemical properties of the NDBs were investigated using FTIR, XRD, TGA, DTA, and AFM to ensure that the novel techniques did not distort the product quality. The NDBs retained their virgin characteristics, namely, their chemical functional groups (FTIR results), crystallinity index (XRD data), thermal stability (TGA and DTA), and ultrastructural features (surface roughness and permeability), as well as their microbial biodegradation ability. Adding PVA enhanced the membrane’s properties except for mass loss, whereby increasing the GA allocation in the NDB blend reduces its mass loss at elevated temperatures. The produced bioplastic membranes showed suitable mechanical properties for food packaging applications and in the pharmaceutical industry for the controlled release of drugs. In comparison to control samples, the separated bacteria and fungi destroyed the bioplastic membranes. *Pseudomonas* spp. and *Bacillus* spp. were the two main strains of isolated bacteria, and *Rhizobus* spp. was the main fungus. The nano-dehydration method gave the best solution for the prompt drying of water-based biopolymers free of manufacturing defects, with simple and easily acquired machinery required for the casting and peeling tasks, in addition to its wonderful biodegradation behavior when buried in wet soil.

## 1. Introduction

Despite petroleum-based polymers’ lower density and greater mechanical characteristics, biobased polymers have gained significant interest due to growing environmental concerns about their sustainability and biodegradability [1,2,3,4,5,6]. Many biopolymers, including poly-lactic acid/poly-lactide, poly_–_3–hydroxy-butyrate, starch, gelatin, alginate, agar agar, guar gum, and GA, have been used in various industrial applications.

GA is a type of edible and natural exudate gum arising from the mature trunks and branches of various acacia species, especially the *Acacia senegal*, *A. seyal*, *A. nilotica*, and *A. mellifera* (family: Fabaceae) [7]. The GA yield can be enhanced by drought conditions, poor soil fertility, and injured or scratched plants to cover the huge demand for domestic and industrial applications due to its water-soluble and polysaccharide nature [8,9,10]. Chemically, GA has hydrophilic carbohydrates (arabinogalactans) as well as hydrophobic proteins (the arabinogalactan–protein complex and glycoproteins) that exhibit various functional properties in food additives [11,12,13,14,15]. It is well known that D-galactose, l-arabinose, L-rhamnose, D-glucuronic acid, and 4–O-methyl-d–glucuronic acid make up the highly branched complexes known as arabinogalactan proteins, along with a minor amount of proteins. While hydrophilic arabinogalactan provides steric and electrostatic stability, this hydrophobic polypeptide chain can tie gum to oil droplets in an emulsion [15]. The benefit of the amphiphilic nature of GA is that it prevents coalescence, which can promote film formation and display steric stabilization. Furthermore, GAs have significant effects on various emulsion factors, including opacity, specific gravity, zeta potential, and surface tension [15]. GA is used primarily in the food, medicinal pharmaceutical, and wood technology industries [15,16,17,18,19,20,21,22,23,24,25,26,27,28,29,30,31,32,33] and as a stabilizer, emulsifier, and thickening agent [15,16,17,18] because of its high hydrophilicity, low fluidic viscosity, good surficial activities, and ability to form a protective film around emulsion droplets [15]. Recently, the use of GA has been extended into the nanotechnology and nanomedicine fields due to its biocompatibility for both in vivo and in vitro applications and its stabilization of nanostructures. GA has been probed for its coating properties and increased biocompatibility of iron oxide magnetic nanoparticles [34,35], gold nanoparticles [36], carbon nanotubes [37], and quantum dot nanocolloids [38].

PVA is a synthetic polymer with a wide range of commercial applications due to its high crystallinity, good mechanical properties, water solubility, and adhesive properties [39,40,41,42]. These applications extend to the industrial, medical, pharmaceutical, and food sectors and include lacquers, resins, surgical threads, food packaging materials [26,29,40], paper coating, textile sizing [43], and coating purposes [44], where they are used in order to enhance the mechanical properties due to natural properties such as compatible structure and hydrophilic properties [45]. Furthermore, it is used as a thermoplastic polymer for living tissues because it is harmless and non-toxic, as well as functioning as a cross-linker and nanofiller [46,47,48]. Furthermore, since PVA has excellent chemical resistance, it is used as a good emulsifier [49]. Practically, PVA is widely blended with other hydrophilic polymers [45], such as GA, to enhance its mechanical properties as well as its biodegradability [42,50,51,52,53,54,55,56]. However, the drawbacks that are associated with the manufacturing of bioplastic materials are the drying process for the cast GA membrane due to the high water content of the parent material, thereby making the entire process time- and cost-intensive. Hence, there is a need to develop an apparatus or method that overcomes the above limitations [57]. The presence of a large number of hydroxyl, carboxylic, and carbonyl groups in the GA/PVA blends makes it a chemical reductant and environmentally benign medium [58]. PVA is easily degradable by biological organisms [39,58], and many microorganisms that are able to degrade PVA and GA have been identified [59,60,61,62].

Different GA composites were prepared and characterized by several researchers for fabricating films and membranes [63,64,65], as well as for the anodes of lithium-ion batteries [66]. In addition, Silvestri et al. [67] produced nanofibers from a blend containing GA (10 wt% solution), graphene oxide (GO), and PVA, while Lubambo et al. [68] obtained nanofibers from guar gum. The processing of GA includes impurity removal, kibbling, granulating, grinding, powdering, acid or enzymatic hydrolysis, clarification and discoloration, centrifugation (10,000–16,000 rpm/min), purification of the ceramic membrane, desalination, and spray drying [69,70,71,72,73].

Several researchers examined the mechanical characteristics of bioplastic materials [74,75,76,77,78,79]. These characteristics play a crucial role in determining the appropriateness of their use in particular applications. Electrospun scaffolds, for instance, should have adequate mechanical characteristics to withstand tension or compression pressures in bone tissue engineering. As assessed by Masti et al. [74], the mechanical properties of their fabricated bioplastic films were assessed by tensile strength (TS) and Young’s modulus (Ym), termed modulus of elasticity (MoE). The addition of gum acacia (GA) to the equal quantity of PVA/CS in the blend film shows improved Ts and Ym. The increase in the Ts and Ym of the blend due to the addition of the GA suggested that interfacial strength (adhesion) could be improved [74,76]. The further addition of GA decreased both Ts and Ym [74,75].

Based on scientific investigations performed by Chougale et al. [76] and Ibrahim et al. [78], their FTIR studies confirmed that due to the crosslinking and intermolecular interactions caused by esterification during the heat treatment in the blend films, there is a significant interaction between each component of their bioplastic mix. Additionally, when compared to the transverse direction, the mechanical performance of the membranes demonstrated an increase in the elasticity modulus in the longitudinal direction from 85 MPa to 148 MPa [78].

A particular category of polymers known as biodegradable polymers [80,81,82,83,84,85,86,87,88,89,90,91,92,93] degrade naturally into byproducts such as gases (CO_2_ and N_2_), water, biomass, and inorganic salts [57] after serving their intended purpose. The abiotic and biotic components of the biodegradation mechanism coexist naturally in the soil [2,6]. Recently, a lot of biodegradable polymers have been created, and some known microbial enzymes can break them down [5]. Numerous microbial communities of bacteria, fungi, and yeasts, including but not limited to Gram-negative species, such as *Escherichia coli* and *Pseudomonas aeruginosa*, and Gram-positive species, such as *Staphylococcus aureus*, can use bioplastic as a nutrition source throughout the biodegradation process [1,3,4,55]. These include *Rhizobium meliloti* [5], *Bacillus* spp. [58], *Pseudomonas* spp., *Aspergillus* spp., *Rhizorpous* spp., *Fusarium* spp., *Penicillium* spp., *Saccharomyces* as yeast [59], and *Elite Aspergillus* [91]. Under aerobic (composting) or anaerobic (landfill) conditions, several petroleum-based polymers are biologically decomposable [47,94]. By combining synthetic and natural polymers, researchers have been able to improve the processing capacity, physicochemical characteristics, and biodegradability of synthetic polymers [1,4,6,50,51,52,53]. For various polymers, including PVA, the rates and environmental factors that influence breakdown might vary [47,60,95,96,97]. Composting can take place under different conditions, including anaerobic environments, underground soil layers, aqueous media, and even in the presence of oxygen.

The aims of the present work were: (a) to invent a more reliable bioplastic membrane that is suitable for different applications; (b) to overcome the casting, drying, and peeling problems of the hydrophilic bioplastic (GA/PVA) blends; (c) to compare the ordinary GA/PVA bioplastic membranes (ADBs) with those synthesized using the novel methods in the present investigation (NDBs); and (d) to study the biodegradation behaviors of the NDBs when buried in wet soil.

## 2. Materials and Methods

**Figure 1:**
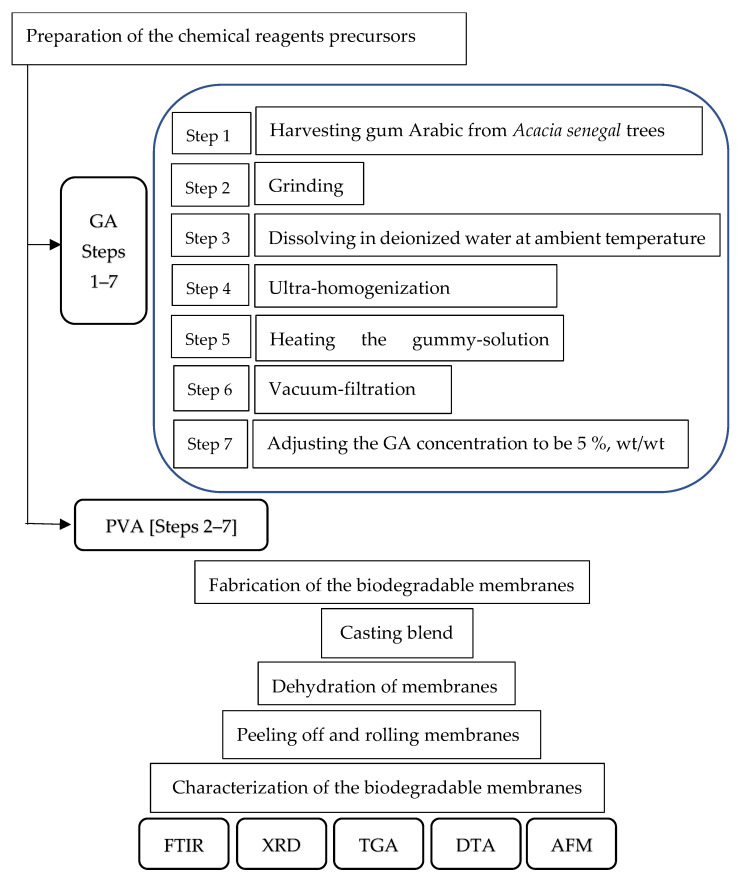
The management plan performed to study the efficiency of the novel manufacturing processes (vibrational casting, nano-dehydration, and self-electrostatic peeling) on the quality of the NDB membranes.

The management plan for the production of novel transparent NDBs is illustrated in Figure 1.

### 2.1. Raw Material

#### 2.1.1. GA

**Figure 2:**
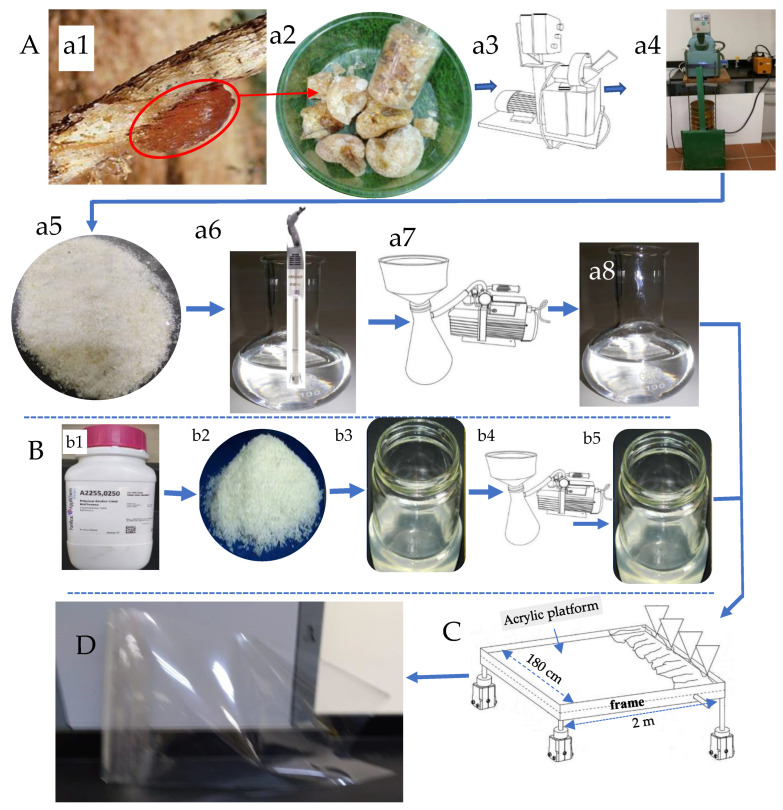
Manufacturing of NDBs: (**A**) Harvesting and processing of the GA principle precursor: (**a1**) hardened sap naturally excreted on a branch of an *Acacia seyal* tree; (**a2**) crude GA granules; (**a3**) mechanical grinder; (**a4**) vacuum-assisted sieving system; (**a5**) uniform powder of crude GA; (**a6**) crude solution of well-dissolved GA; (**a7**) vacuum-filtration device; and (**a8**) clear vacuum-filtered solution. (**B**) PVA-modifier precursor: (**b1**) analytical-grade bottle; (**b2**) powder form; (**b3**) crude solution of well-dissolved PVA; (**b4**) vacuum-filtration device; and (**b5**) clear vacuum-filtered solution; (**C**) VFHF device; (**D**) the NDB final product.

GA (~Mw: 1.827 × 106 g/mol) was harvested from the trunks and branches of *Acacia seyal* trees (Figure 2(a1)) habituated at Hada Al-Sham (about 120 km apart from Jeddah), Saudi Arabia. As shown in Figure 2A, the naturally hardened sap excreted on a branch of an *Acacia seyal* tree was collected after tapping the woody tissues of the tree and making incisions (60 cm × 5 cm). (Figure 2(a1)) and cured into crude granules (Figure 2(a2)). The solid granules were ground using a mechanical grinding machine (Figure 2(a3)), passed through a standard 60 mesh sieve, and retained particles of 80 mesh size (60/80 mesh) using a vacuum-assisted sieving system (Figure 2(a4)). About 50 g of air-dried uniform GA powder (Figure 1(a5)) was dissolved in one liter of deionized water at ambient temperature (25 °C) and heated up to 80 °C with continuous stirring until all particles were completely dissolved (Figure 1(a6)). Removing the insoluble components of the resultant solution was achieved via vacuum filtration (Figure 2(a7)) to obtain the clear GA precursor at a concentration of 5% *wt*/*wt* (Figure 2(a8)).

#### 2.1.2. PVA

PVA was used as a modifier precursor to synthesize the NDB, along with GA. PVA used in this investigation (Figure 2B) was of ACS reagent quality (Figure 2(b1)), Mw 88,000 Da, and 88% deacetylated. About 50 g of PVA crystals (Figure 2(b2)) were dissolved in one liter of deionized water to obtain a crude solution (Figure 2(b3)) as a result of heating under continuous stirring at 80 °C until complete clearance and subsequently vacuum-filtered (Figure 2(b4)) to obtain the clear PVA precursor at a concentration of 5% *wt*/*wt* (Figure 2(b5)).

### 2.2. Preparation of the Bioplastic Blends

The practical procedure used for the novel casting of the bioplastic blends is presented in Figure S2. Six different bioplastic blends of GA and PVA in different ratios were prepared by mixing their aqueous solutions (5% *wt*/*wt* each) under continuous and calm stirring until the solution became completely homogenous (see Figure 2). It is crucial to stir slowly and carefully to prevent the addition of too many air bubbles to the solution, which can result in aeration defects in the final membranous product.

### 2.3. The Casting Platform

The practical procedure used for casting the bioplastic blends is presented at Figure S1 (see in Supplementary Materials). After mixing known concentrations of GA and PVA to obtain a homogenous clear solution, the blend was casted on a novel platform made up of poly-(methyl methacrylate) abbreviated as PMMA and known to be acrylic panel. We chose this material in the current experiment as an ideal casting platform for polymers, particularly water-based ones. This selection of PMMA was due to its non-stick surface, which can help the bioplastic blend be peeled easily after drying and curing [57]. The PMMA panel was irradiated using UV-waves to activate its electrostatic charges and rising its temperature up to 50 °C.

As shown in Table S1 and Figure 2C and Figure S2, the PMMA panel (180 cm in width, 2 m longitudinally, 8 mm in thickness) was fixed on the upper panel of the casting table. Furthermore, adjacent strips of PMMA can be arranged to cover a movable belt that may be used as a casting surface [57]. Before pouring the bioplastic blind onto the PMMA’s substrate, vibrational forces were generated by using suitable solenoids to ease spreading the blend in a definite velocity over the worm casting platform. After that, a mild air drying of the molten polymeric membrane was applied in order to thicken its viscosity.

### 2.4. Casting the Bioplastic Blends

**Figure 3:**
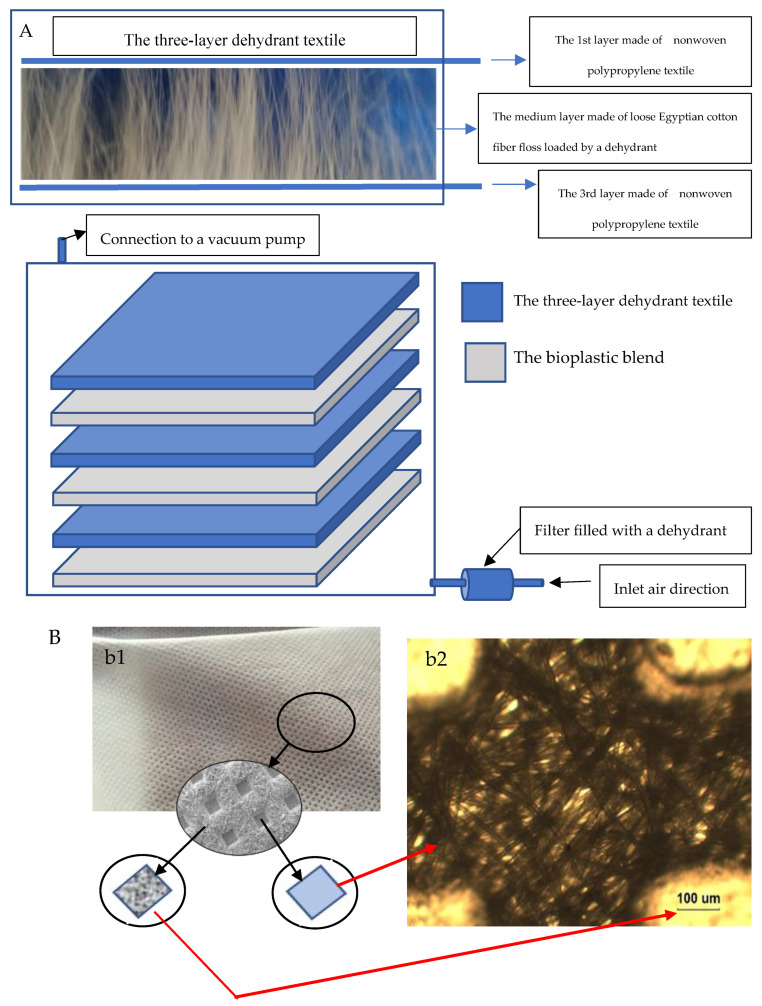
The novel nano-dehydration technique: (**A**) the stratified air-dryer apparatus, and (**B**) the non-woven textile of polypropylene: (**b1**) an optical image, and (**b2**) microscope image (40×) according to Hindi and Albureikan [57].

After obtaining complete homogeneity for the biopolymer blend, the bubble-free ternary blend solution was poured onto a clean acrylic panel. This panel is the surficial layer of the VFHF device (Figure 2C and Figure S3), with a prominent frame where it is necessary to adjust the initial thickness of the bioplastic membrane to determine its final thickness. After that, the cast blend was allowed to dry at ambient temperature using the novel stratified nano-dehydrator (SND) apparatus [57,98,99], as shown in Figure 3.

The novel VFHF device (Figure 2C and Figure S2, see in Supplementary Materials) features both the ease of casting the blend and peeling the membranes with a constant thickness free of air bubbles. For manufacturing a NDB, the blend was poured after adjusting the slope angle of the acrylic ground template (Figure 2C and Figure S2) to a slight angle (about 15°) in order to accelerate the blended fluid movement. The slow motion of the blend protects its matrix from forcing more bubbles inside it. After the blend reached the opposite side of the template, the slope angle was re-adjusted to zero degrees to ensure exact horizontality in order to obtain identical thicknesses. It is worth mentioning that the thickness of the bioplastic sheets was controlled by two critical actions: (a) pouring a definite quantity of blend solution onto the same template area and (b) accurate adjustment for the viscosities of these blends [57].

In order to obtain a gentle, steady, and efficient flow for the viscous bioplastic blend, each of the four legs of the VFHF was fixed with a vibrating magnetic solenoid (a Kendrion OSR series shaker) that was designed with two excitation coils to generate a harmonious vibrating movement in the blend [57]. The magnetic vibrating system (Figure S3B) consists of a permanent magnet at the bottom, connecting the magnetic body’s two halves and two excitation windings. The body to be vibrated, which serves as the armature, closes the magnetic circle through an air gap. A steady pulling force between the magnetic body and armature is produced by the permanent magnet that is integrated into the magnetic body, biasing the system. The alternating electromagnetic field’s force effect superimposes itself onto the permanent magnet’s force effect when an AC voltage is delivered to the excitation coil. The fully encapsulated bobbin and coils achieve reliable long-life service and maintenance-free operation. In addition, OSR shaker solenoids are not susceptible to dust or moisture when operational under rough or adverse conditions [57]. It is worth mentioning that a permanent magnetic attachment serves to mount the OSR shaker solenoid freely and that it is detachable from the vibrating surface. Angle mounts permanently fix the OSR shaker solenoid to a vibrating surface. In addition, phase angle controllers were installed separately for the fine adjustment of vibration through alternating or direct current (via an integrated one-way rectifier), and they can be DIN-rail mounted within cabinets with minimal space.

### 2.5. Drying the NDBs

#### The Stratified Nano-Dehydrator (SND)

The most critical problem concerning the manufacturing of bioplastic sheets is their drying process, where it is crucial to obtain high-quality products. This problem arises from the highly hydrophilic nature of bioplastic sheets blended with mixtures of hydrophilic GA and PVA in different ratios.

The SND was invented to accelerate the dehydration process of bioplastic sheets [57]. As shown in Figure 3, it consists of three stratified and perforated acrylic panels (poly-(methyl methacrylate)). These panels were arranged in a vertically alternating pattern, with three sub-layers consisting of dehydrant-loaded fibrous materials. Each sub-layer constitutes two non-woven polypropylene textiles, restricting an intermediate net of loosened Egyptian cotton floss (ECF). All fibrous materials constructing the sub-layer are saturated with a strong dehydrant, such as phosphorus pentoxide (P_4_O_10_), the most highly preferred dehydrant reagent, rather than calcium chloride, magnesium sulfate, aluminum oxide, lithium aluminum hydride, metallic sodium, or silica gel. Removing water by P_2_O_5_ was found to be more complete, quickly, effectively, and at a faster rate than many other dehydrants.

This cellulosic material was selected for this task due to its high content of alpha cellulose, well-known for its high hydrophilicity, which is essential to attaining good affinity to both moisture molecules as well as dehydrant crystals. The manner of loading dehydrant onto the cellulosic fibers can be summarized as follows: (1) air-drying of cellulosic fibers used as a core skeleton of the SND; (2) preparing dehydrant-saturated solution; (3) soaking cellulosic fibers in the salt-saturated solution via vacuum forces to ensure complete immersion and saturation of the fiber cells and penetration of the salt solution into the cell cavities, as well as the cell wall, through the internal border pits of the cellulosic fibers; (4) discarding excess salt solution and drying the cellulosic fibers by air-drying and finally oven-drying at 80 °C ± 5 °C for 2 h; and (5) this medium layer was inserted between the first and third layers, which were made of non-woven polypropylene textile. The edges of the outer layers were welded thermally due to the nature of polypropylene as a thermoplastic material. Furthermore, the three-layer textile was reinforced upon stitching using a sewing machine.

### 2.6. Peeling off the Bioplastic Membranes

A self-electrostatic charged-template (SECT) made of poly-(methyl methacrylate) was used as a casting platform, which made peeling the final product membranes easy due to its non-stick behavior [57]. It can be used as a table cover termed an acrylic platform (Figure 2C) or as a covering layer (by coating or adhesion) for a movable belt to give production continuity.

The resulting transparent bioplastic sheet (Figure 2D) was peeled slowly and accurately away from the PMMA platform, rolled, and stored under dry circumstances until used.

### 2.7. Characterization of the Bioplastic Membranes

The values of the different physical and chemical properties of the bioplastic sheets were calculated as presented in Table 1.

#### 2.7.1. FTIR

Using a Bruker Tensor 37 FTIR spectrophotometer, the chemical constituents and functional groups of the six bioplastic membranous samples were investigated. The samples were combined with KBr at a ratio of 1:200 *wt*/*wt* and compressed under vacuum to form pellets after being oven-dried at 100 °C for 4–5 h. The materials’ FTIR spectra were captured between 400 and 500 cm^−^^1^ in transmittance mode.

#### 2.7.2. X-ray Diffraction (XRD)

An XRD 7000 Shimadzu diffractometer (Kyoto, Japan) was used to determine the XRD spectra of the six bioplastic sheets. An anode generator, a copper target, and a wide-angle powder goniometer are all parts of the system. Measurements were performed with the aid of CuK radiation arising at 30 kV and 30 mA. The Kα1 (0.15406 nm) and Kα2 (0.15444 nm) components of the CuK radiation are present in the resulting XRD data.

A single-channel analyzer was used to extract the data resulting from the semiconductor detector. The reception slit was 0.15 mm at the same radius, and the divergence and scatter slits were each 10 m wide. Several droplets of diluted amorphous glue were used to mount dried bioplastic sheets (weighing around 0.5 g) onto a quartz platform. Each sample was scanned in the 2θ° range between 5° and 40°. Every experiment was run in reflection mode, with increments of 0.05° and a scan speed of 4°/min [57,98].

The crystallinity index (CI) was computed by dividing the diffractogram area of crystalline cellulose by the entire area of the original diffractogram after smoothing the resulting crystalline peaks from the diffraction intensity profiles. Using Microsoft Excel (USA), the area under the curve was calculated by adding adjacent trapezoids [98,99,100,101,102,103].

#### 2.7.3. Thermal Analysis

Since DTA typically complements TGA with information on phase transitions [93], the TGA and DTA of each blend were conducted simultaneously. These characterizations were carried out for TGA and DTA for all six bioplastic blends [57,98,99,101] using a Seiko and Star 6300 analyzer, Central Laboratory, Faculty of Science, Alexandria University, Egypt.

Using a heating rate of 20 °C/min under a nitrogen atmosphere, heating scans were carried out from 30 °C up to the final maximum temperature of 450 °C [57,98,101].

Determination of the NDB mass loss estimated from the TGA curves was achieved as illustrated in Table 1.

#### 2.7.4. Surface Topography (ST)

Atomic force microscopy (AFM) was used to examine the surfaces of the six NDBs in order to observe the full 3D membranous surface structures down to the nanometric scale. Four distinct characteristics of the NDB were revealed by AFM [57,98]: surface roughness (SR), nanometric particle size (NPS), pore diameter (PD), and void volume (VV).

### 2.8. Mechanical Properties of the Bioplastic Membranes

The stress–strain behavior of the six bioplastic membranes, else ADB NDB, was measured using The Instron universal testing machine, model 1193, Instron Co., Ltd., London, UK, with a 200 N-load cell according to the ASTM D–882 standard test, 1989. The bioplastic membrane samples were rectangular-shaped (2.5 × 10 cm) for each of the ADBs and NDBs membranes. The device with two metallic grips was installed to hold the test sample at both ends. The starting grip separation for all samples was 50 mm, and the upper grip was extended at a constant rate of 50 mm per minute while the lower grip remained stationary. An automatic speed controller was fitted to the electrically powered machine to maintain the upper grip’s speed. The ambient temperature was used for all measurements. From the plot of stress–strain curves, the UTS, MoE, and EaB for each film were estimated, as illustrated in Table 1.

The mechanical properties, namely, ultimate tensile strength (UTS) in MPa, modulus of elasticity (MoE) in MPa, and elongation at failure (EaF) as a percentage, were calculated.

#### 2.8.1. Ultimate Tensile Strength (UTS)

The UTS shows the film’s maximum allowable tensile stress [74,75,76]. The UTS property of the bioplastic sample was calculated by dividing the maximum load causing the film failure by the cross-sectional area of the film, as explained in Table 1 [74,75,76].

#### 2.8.2. Modulus of Elasticity (MoE)

The membrane’s MoE is a reliable indicator of its stiffness [74,75,76]. The MoE was computed by dividing the length of the membrane sample at yield by the stress at yield, as expressed in Table 1.

#### 2.8.3. Elongation at Failure (EaF)

The EaF was calculated by dividing the elongation at failure of the sample by the initial gauge length, as shown in Table 1 [74,75,76].

### 2.9. Microbial Biodegradation

Microbial biodegradation was assessed to investigate the microbes’ capacity to break down the buildup of bioplastic in soil. Upon calculating the percentage of biodegradation (weight loss), counting the microbe population isolated from the surfaces of bioplastic sheets, and evaluating the various morphological changes in these surfaces as a result of degradation, it was possible to determine the amount of biodegradation [98,99].

The soil was collected from the Agricultural Research Station (ARS) of King Abdullaziz University’s Faculty of Environmental Sciences in Hada Al–Sham and was used to bury the bioplastic samples. The location is situated 240 m above sea level, around 120 km to the northwest of Jeddah (N = 21°48′3″, E = 39°43′25″). The pH of the soil at the site ranged from 7.1 to 7.9, along with low levels of CaCO_3_, organic matter, and cation exchange capability [104,105].

#### 2.9.1. Sample Preparation and Soil Burial Studies

The various bioplastic sheets were shredded into 2 cm × 2 cm pieces and buried 10 cm deep in 100-gram wet soil boxes. Before being buried in the ground, each bioplastic piece was weighed (0.040–0.038 g). By adding sterile water to the soil samples to counteract water evaporation, the moisture content of the soil samples was kept constant [98].

Each sample box had a hole at the bottom for the excess water to drain through. After 30 and 60 days, soil samples were carefully removed, and the weight loss was calculated in order to separate, count, and compare the microbial community, as well as speculate on microbial morphology changes as a result of degradation [98,99,106].

#### 2.9.2. Isolation and Counting of Microbial Communities

About 95 mL of sodium pyrophosphate solution at 0.1% *wt*/*v* was used to suspend one gram of soil collected from the surface of the bioplastic sheets from each sample. The samples were left to stand for 30 min. Then, using the serial dilution method, the supernatant was divided among six tubes, and one milliliter (mL) of each dilution was plated on nutrient agar medium, NA (Oxoid), for the isolation of bacteria, while potato dextrose agar medium, PDA (Oxoid), was used for isolation of fungi.

Finally, in order to determine the colony-forming units (CFUs), plates were incubated at 30 °C and pH 7 for 720 h (for bacteria) and at 25 °C and pH 5 for 1440 h (for fungi). Based on their cultural and physical characteristics, microorganisms were separated and identified using conventional assays [1,57,98,99].

### 2.10. Statistical Design and Analysis

Various properties of the six bioplastic membranes synthesized from the aqueous solutions of GA and PVA were assessed using a randomized complete block design. According to El–Nakhlawy [107], a statistical analysis of the obtained data was carried out using the analysis of variance approach and the least significant difference test (LSD) at 0.05.

## 3. Results

### 3.1. Chemical and Physical Properties of the Bioplastic Membranes

#### 3.1.1. FTIR

**Figure 4:**
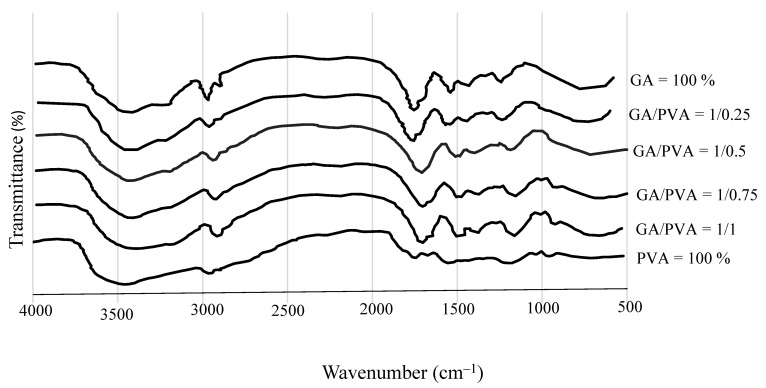
FTIR spectra of the six transparent nanodehydrated bioplastic (NDB) membranes over the wavenumber range of 4000 to 500 cm^−1^, fabricated from various gum Arabic (GA)/polyvinyl alcohol (PVA) blends.

FTIR spectroscopy was used to determine the chemical functionality of the bioplastic sheets (the nanodehydrated membranes (NDMs), as illustrated in Figure 4. The spectra of the resulting NDBs exhibited chemical group absorption bands that were typical of the gummy products made from both GA and PVA in different ratios. The absorption bands spanned an area between 500 and 4000 cm^−1^. Several chemical groups were precisely found at the following wavenumbers: 900–1250, 1426, 1402, 1625.4, 1627.4, 1430, 1436.91, 1437, 1641, 1047, 2800–3000, 2885, 2910.87, 2939, 3000, 3261, 3416, 3000, and 3600 cm^−1^ [57,98,99,101,108,109,110,111].

#### 3.1.2. XRD

**Figure 5:**
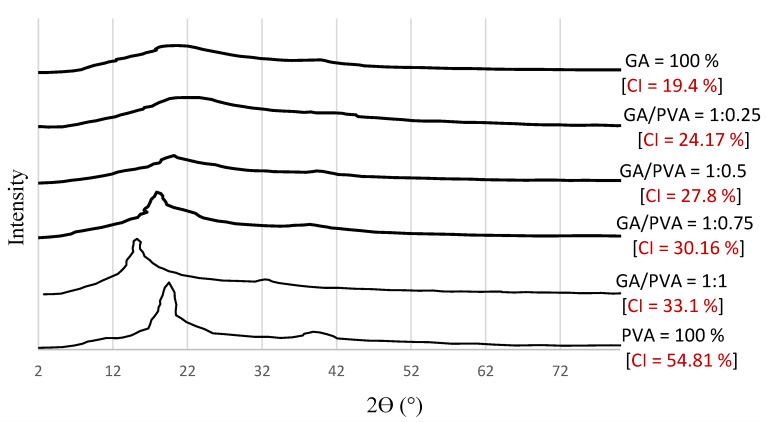
XRD diffractogram spectra of the six transparent nanodehydrated bioplastic (NDB) membranes over a wavenumber range of 4000 to 500 cm^−^^1^, fabricated from various gum Arabic (GA)/polyvinyl alcohol (PVA) blends, showing the crystallinity index (CI) values.

Figure 5 displays the XRD patterns of the six bioplastic membranes. The maximum intensity of the GA-broad diffractogram was obtained at 2θ = 20°, which confirms the amorphous nature of the gum Arabic [13]. Moreover, a typical peak for pure PVA, a semi-crystalline polymer, was visible at 2θ° = 19.9°. With the increase in PVA allocation in the blend, the crystallinity index values increased (Figure 5).

As demonstrated in Figure 5, the CI values of the NDBs were found to increase from 19.4% (for pure GA) to 54.81% (for pure PVA). Accordingly, it is clear that the increase in CI of the bioplastic blends can be attributed to an increase in the PVA allocation in the blend.

#### 3.1.3. TGA

**Figure 6:**
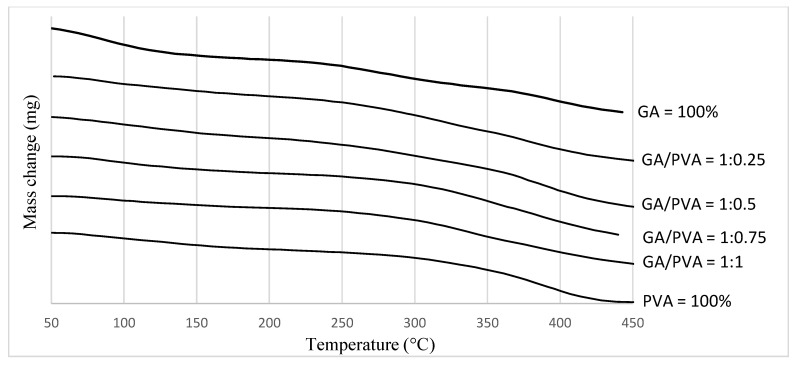
Thermogravimetric analysis (TGA) thermogram spectra of the six bioplastic membranes (NDBs) in the wavenumber range of 4000 to 500 cm^−^^1^, fabricated from various gum Arabic (GA)/polyvinyl alcohol (PVA) blends.

The TGA results are presented in Figure 6 and in Table 2 and Table S2. The mass losses of the six NDBS samples were focused on eight temperature regions, namely, 50–100 °C, 100–150 °C, 150–200 °C, 200–250 °C, 250–300 °C, 300–350 °C, 350–400 °C, and 400–450 °C (Table 2 and Table S2; Figure 6).

The thermal degradation of the samples increased with rising temperatures for all six bioplastic blends, according to a comparison of mass losses between temperature zones (at the same bioplastic blend ratio).

Comparing the mass losses within the temperature zone meant studying the differences between bioplastic blend ratios in the same temperature zone. It is clear from Table 2 and Table S2 and from Figure 6 that at lower temperatures (≤150 °C), PVA lost more weight (5.69% and 8.98% for 50–100 °C and 100–150 °C zones, respectively) than GA (13.68% and 11.11%) in the same temperature zones. On the other hand, at higher temperatures, this trend was reversed, whereby PVA lost more weight (79.01% and 58.8% for the 400–500 °C and 450–500 °C zones, respectively) than GA (28.6% and 15.6% for the same zones, respectively).

#### 3.1.4. DTA

**Figure 7:**
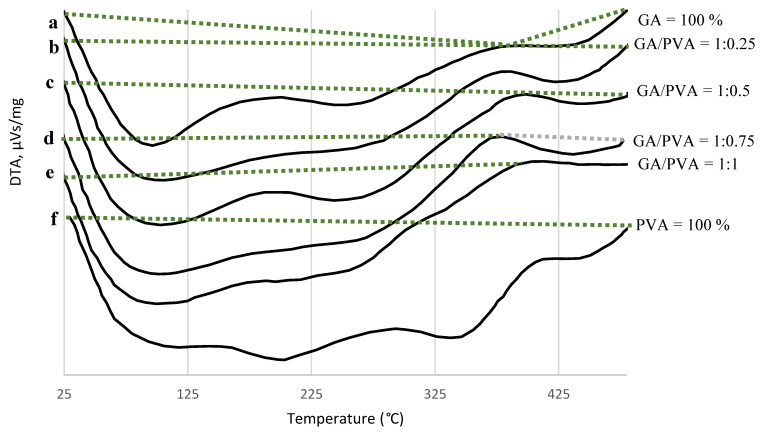
Thermograms of differential thermal analysis (DTA) of the six nanodehydrated bioplastic (NDB) membranes over a wavenumber range of 4000 to 500 cm^−1^, fabricated from various gum Arabic (GA)/polyvinyl alcohol (PVA) blends.

The DTA analysis findings of the six nanodehydrated NDBs are presented in Figure 7 and Table 3.

Examining Figure 7 and Table 3, the NDB thermograms were found to be divided into two sets representing the bioplastic blends, namely, the single-phase and double-phase thermograms. The single-phase thermogram is composed of one endothermal phase, namely, curve ‘b’ (GA/PVA of 1:0.25), curve ‘e’ (GA/PVA of 1:1), and curve ‘f’ (PVA = 100%). On the other hand, the double-phase thermogram is differentiated into two distinct regions (endothermic and exothermic), namely, curve ‘a’ (GA = 100%), curve ‘c’ (GA/PVA of 1:0.5), and curve ‘d’ (GA/PVA of 1:0.75).

For more information, see Table 3. It is evident that the temperature ranges of each thermogram and the absolute values of the heat change (HC) values for the endotherms (16 Vs/mg–52.4 Vs/mg) were larger than those for the exotherms. Additionally, among the other bioplastic blends, the pure PVA endotherm absorbed the most energy (2119.7 Vs/mg), but GA had the lowest value of heat change (−1017.3 Vs/mg).

### 3.2. Ultrastructure of the Bioplastic Membrane

#### 3.2.1. Surface Roughness (SR) and Particle Size (PS)

**Figure 8:**
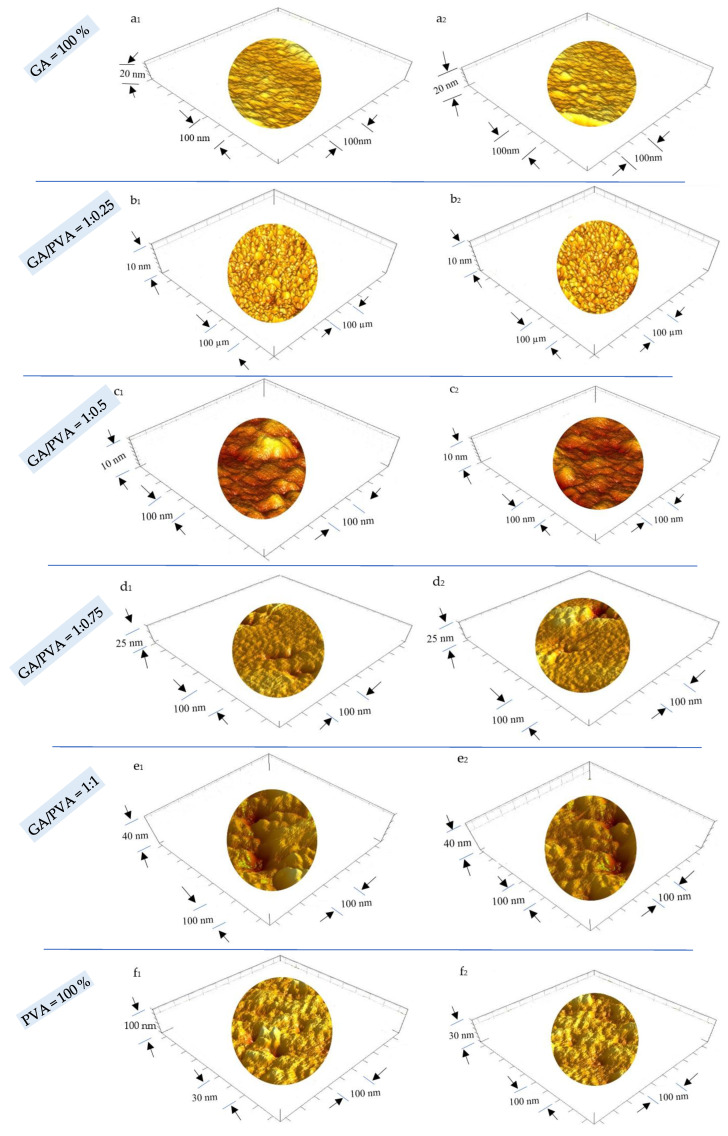
AFM images of surface roughness of the bioplastic membranes blended from gum Arabic (GA) and/or polyvinyl alcohol (PVA) precursors in different ratios: (**a_1_**–**f_1_**) air-dried membrane (ADB) and (**a_2_**–**f_2_**) nanodehydrated bioplastic membrane (NDB).

**Figure 9:**
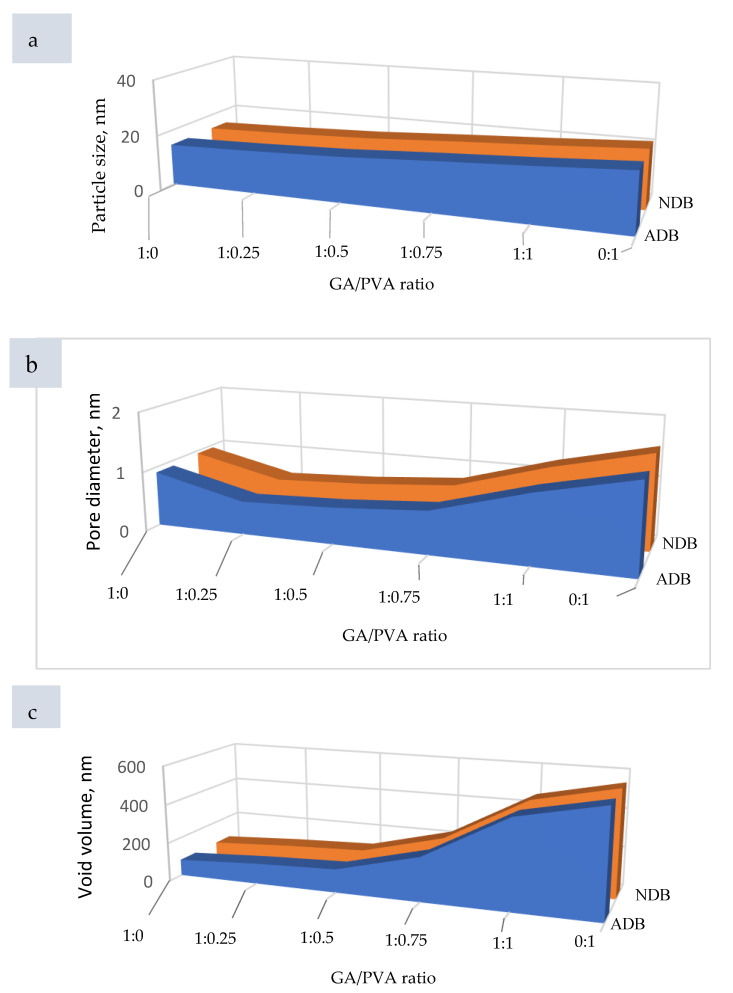
Ultrastructure features of the air-dried membrane (ADB) and the nano-dehydrated-bioplastic membrane (NDB): (**a**) particle size, (**b**) pore diameter, and (**c**) void volume as affected by different allocations of GA and PVA (GA/PVA blends).

In order to confirm the similarity between the ultrastructure features of ADBs and NDBs, the RS was investigated via atomic force microscopy (AFM) and is presented in Figure 8 for each of the six bioplastic blends. For clear, the GA/PVA blends’ membranes dried by air are presented in Figure 8(a_1_–f_1_), while those for the NDBs are found in Figure 8(a_2_–f_2_). Since the nanometric PS is known to be intimately related to the SR, its data shown in Table 4 and Figure 9a are useful to shed an excess of light over the SR of the bioplastic membranes.

Paying attention to the AFM’s images (Figure 8) revealed to that the RS was increased from the 1st blend ratio (GA = 100 %) until reaching the 6th blend ratio (PVA = 100 %). This finding can be attributed to the higher PS value of the PVA comparing to that for the pure GA as clear when speculating the gradual increasing of the PS values along with the six bioplastic blends. However, this trend was found to be similar for the ADBs and NDBs.

Regarding to the PS’ results, see Table 4, statistical comparisons were performed between the membranes (ADEs and NDBs) as well as within the membranes (between the GA/PVA blends, namely, 1/0, 1/0.25, 1/0.5, 1/0.75, 1/1, and 0/1). Comparing membranes, there was no statistical difference between the ADBs and NDBs concerning their particle size (13.57 and 14.77 nm, respectively). On the other hand, comparing the blend ratios within the membrane (Table 4, Figure 8(a_1_,a_2_) and Figure 9a) revealed that the GA membrane (GA = 100%) had the lowest PS for each of the means (13.57 nm), with a maximum value (55.44 nm). Furthermore, PVA sheets had the highest PS values (20.34 and 89.75 nm for the mean and maximum values, respectively). In between, increasing the PVA concentration in the bioplastic blends increased the PS gradually (Table 4 and Figure 8(f_1_,f_2_) as well as Figure 9a).

#### 3.2.2. Pore Diameter (PD) and Void Volume (VV) of the NDB Membranes

**Figure 10:**
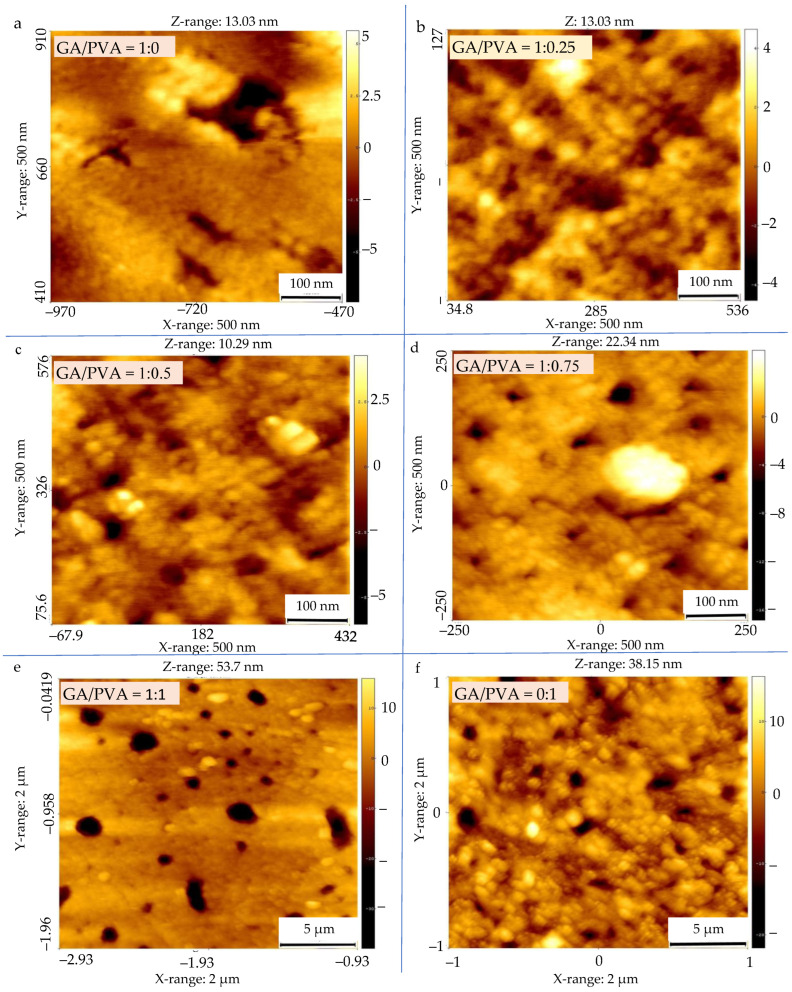
Permeability of the nanodehydrated bioplastic membranes blended from gum Arabic (GA) and polyvinyl alcohol (PVA) precursors in different ratios of GA/PVA: (**a**) 1/0, (**b**) 1:0.25, (**c**) 1:0.5, (**d**) 1:1.75, (**e**) 1:1, and (**f**) 0/1, respectively (AFM images).

Data produced for PD are presented in Table 4 and Figure 9b, Figure 10 and Figure S4, while Table 4 and Figure 9c represent the VV’s results. The same ascending trend was noticed for both PD and VV regarding their influence, with an increase in the PVA allocation in the ADBs as well as the NDBs. The PD of the ADB increased from 0.91 nm to 1.485 nm for the GA/PVA ratios of 1/0 and 0/1, respectively. In addition, the VV of the ADB increased from 83.24 nm^3^ to 548.95 nm^3^ for the GA/PVA ratios of 1/0 and 0/1, respectively.

In addition, there was no statistical difference between ADBs and NDBs in their PS, PD, and VV; consequently, there is no evidence that the novel procedures used in the bioplastic membrane preparation alter their parent ultrastructure.

### 3.3. Mechanical Properties of the Bioplastic Membranes

**Figure 11:**
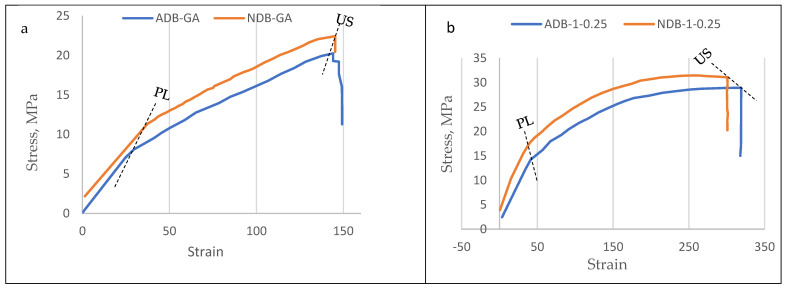

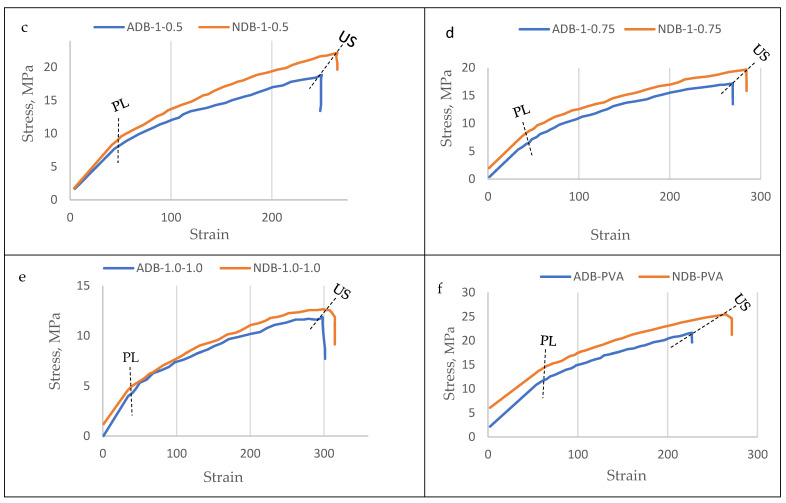
Stress–strain graphs of the six bioplastic membranes fabricated from gum Arabic (GA) and polyvinyl alcohol (PVA) with different ratios for each of the air-dried (ADB) and nanodehydrated (NDB) membranes showing proportionality limit (PL) and ultimate stres (US).

**Figure 12:**
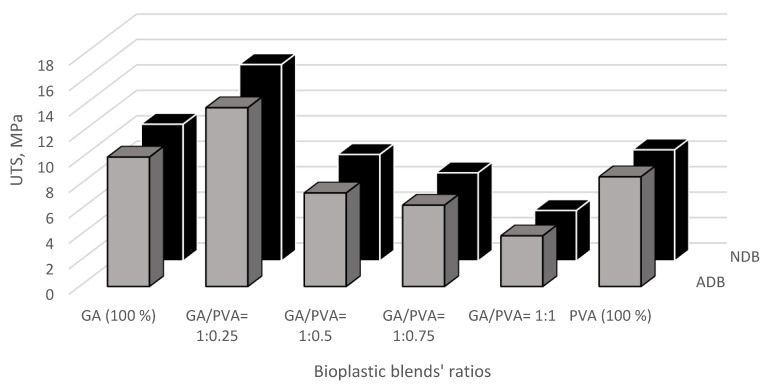
Ultimate tensile strength (UTS) of the six bioplastic membranes fabricated from gum Arabic (GA) and polyvinyl alcohol (PVA) with different ratios for each of the air-dried and nanodehydrated membranes.

**Figure 13:**
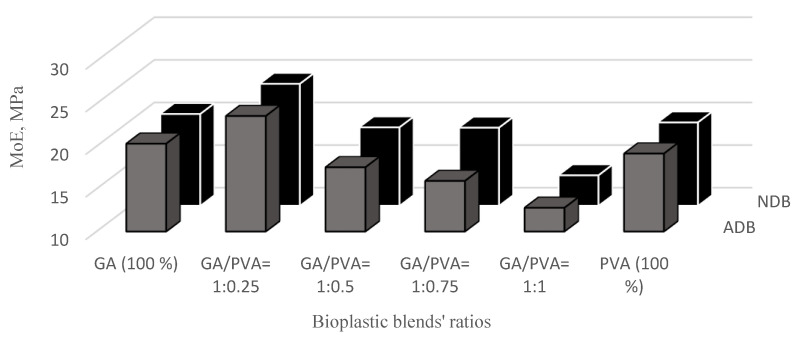
Modulus of elasticity (MoE) of the six bioplastic membranes fabricated from gum Arabic (GA) and polyvinyl alcohol (PVA) with different ratios for air-dried (ADB) and nanodehydrated membranes (NDB).

**Figure 14:**
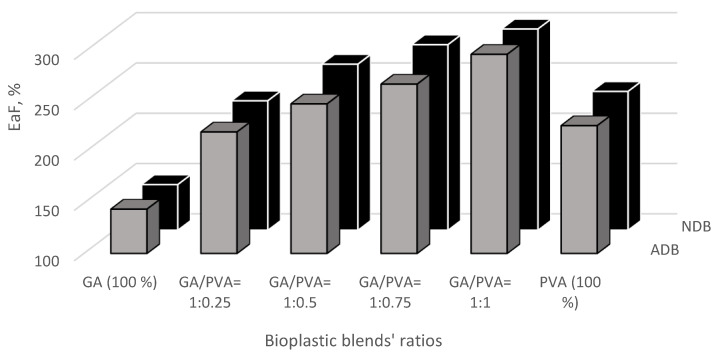
The percentage of elongation at failure (EaF) of the six bioplastic membranes fabricated from gum Arabic (GA) and polyvinyl alcohol (PVA) with different ratios for each of the air-dried (ADB) and nanodehydrated (NDB) membranes.

The results of the mechanical investigation of gum Arabic/poly (vinyl alcohol)/blend films were presented in Figure 11, Figure 12, Figure 13 and Figure 14. Stress–strain curves of the six bioplastic membranes fabricated from GA and PVA are shown in Table 5 and Figure 11. For more specification, ultimate tensile strength (UTS), modulus of elasticity (MoE), and elongation at failure (EaF) are clear in Figure 12, Figure 13 and Figure 14, respectively. Concerning Figure 11, it is worth mentioning that the disappearance of air bubbles in the bioplastic membranes as well as their clear transparency suggested the compatibility of the well-blended components [76] and consequently enhanced their mechanical properties. Simplifying illustration of the Figure 11, it presents the stress in mega Pascal units that affects the rheological endurance of each of the six bioplastic membranes expressed by the strain properties of the six blends. It is worth of mentioning that the mechanical relationship between stress and strain was determined for each of the ADB and NDB membranes.

As clear from Table 5 and Figure 11, the plotted stress-strain curves for the six blended membranes were differed concerning to their proportionality limit (PL) and ultimate strength (US). Regarding to sub-graphs of the bioplastic membranes in Figure 11a–f, both ADB (the red curve) and NDB (the blue curve) are similar in their ascending trend starting from the PL level up to the US. This behavior means that each membrane, else ADB or NDB was stressed through two stages: (1) in the 1st one, the stress was increased from zero up to the PL level, and (2) through the 2nd stage, each membrane transitioned from elastic to plastic nature as the load was increased from the PL up to the maximum load resulting the US stage.

Regarding to sub-graphs of the bioplastic membranes in Figure 11a–f, both ADB (the red curve) and NDB (the blue curve) are similar in their ascending trend starting from the PL level up to the US. This behavior means that each membrane, else ADB or NDB was stressed through two stages: (1) in the 1st one, the stress was increased from zero up to the PL level, (2) through the 2nd stage, each membrane transitioned from elastic to plastic nature as the load was increased from the PL up to the maximum load resulting the US stage.

Regarding to proportionality limit (PL) of the bioplastic membranes, it is higher for the NDB than that for the ADB for all the six blend ratios. This indicates that the NDB membranes has higher elasticity endurance compared to their analogous membranes.

#### 3.3.1. Ultimate Tensile Strength (UTS)

The observed curves of the UTS for the six polymeric blend membranes are presented in Figure 12.

The bioplastic membrane with the blend ratio of GA/PVA = 1:0.25 was found to have the highest UTS values (14.05 MPa and 15.44 MPa for ADB and NDB, respectively). It can also be seen from Figure 12 that the GA had a higher UTS’ mean value than that for PVA (8.62 MPa and 8.74 MPa for ADB and NDB, respectively).

For more illustration, tensile stress increased as the GA content decreased from GA=100 % which has no PVA content (10.17 MPa, 10.75 MPa for ADB and NDB, respectively) up to the membrane with GA/PVA = 1:0.25 (14.05 MPa, 15.44 MPa for ADB and NDB, respectively). After that, the UTS decreased gradually with the consequent decrease in GA, thus increasing the PVA allocations in the bioplastic blend. Moreover, no significant difference was detected between the bioplastic membranes dehydrated by ordinary and nano-techniques (ADB and NDB) for all six bioplastic blends (Figure 12).

#### 3.3.2. Modulus of Elasticity (MoE)

Regarding the MoE graph presented in Figure 13 for the six bioplastic blends, the blend ratio of GA/PVA = 1:0.25 had the highest MoE values (14.05 MPa and 15.44 MPa for ADB and NDB, respectively). Moreover, GA was slightly higher in its MoE mean value than that for PVA (19.14 MPa and 17.79 MPa for ADB and NDB, respectively).

Also from Figure 13, it is worth mentioning that both graphs of ADB and NDB are similar in their trend concerning to the MoE curve, in which they increase with decreasing GA’s and increasing PVA’s allocation in the blend up to the blend ratio of 1:0.25. For more illustration, the mean value of the MoE was increased from the GA, 100%, and zero-allocation of PVA (20.31 MPa and 20.77 MPa for ADB and NDB, respectively) up to the membrane with GA/PVA = 1:0.25. After that, the UTS was decreased by decreasing the GA and increasing the PVA allocations in the bioplastic blend. Moreover, MoEs’ mean values were found to be statistically similar concerning the bioplastic membranes dehydrated by ordinary and nano-techniques (ADB and NDB) for all six bioplastic blends (Figure 13).

#### 3.3.3. Elongation at Failure (EaF)

The EaF data presented in Figure 14 indicates that adding PVA amounts to the bioplastic blends shows a significant increase in the EaF of the produced membranes for the GA/PVA’s blinding ratios of 1:0.25, 1:0.5, 1:0.75, and 1:1. In addition, it can also be seen from Figure 14 that the PVA had higher EaF’s mean values (227.09% and 237.91%, for ADB and NDB, respectively) than those for GA (144.04% and 145.25%, for ADB and NDB, respectively). Moreover, there are no significant differences in the UTS belonging to the bioplastic membranes dried by means of ordinary and nano-dehydration methods (ADB and NDB) for all six bioplastic blends (Figure 14).

### 3.4. Bacterial and Fungal Biodegradation

The microbial communities for the initial soil samples as well as the buried bioplastic sheets were found to be different in number and species (Table 6). Depending on the type of buried membrane, different types of bacteria and fungi were found. *Pseudomonas* spp. [112,113], *Bacillus* spp. [58,112,113], *Aspergillus* spp. [114], and *Penicillium* spp. [112,115] were the predominant species for the buried PVA. *Bacillus* spp. [59], *Pseudomonas* spp., *Aspergillus* spp., *Rhizorpous* spp., *Fusarium* spp., *Penicillium* spp., and yeast *Saccharomyces* [59] were additional important species for the buried GA.

Moreover, the (GA/PVA = 1:1) bioplastic blend’s microbial populations included *Bacillus* spp. [58,116], *Pseudomonas* spp., *Aspergillus* spp., *Rhizorpous* spp., *Fusarium* spp., and *Penicillium* spp. In addition, more fungal species than bacteria were found, which is consistent with the findings of Hindi et al. [98], who discovered that fungal isolates had a higher ability to use the sheets as growth substrates than bacteria.

Table 6 contains information about the colony-forming units (CFUs) of different microbial species. The total counts of bacteria, fungus, and yeast were determined to be 2.28 × 10^5^ and 1.88 × 10^3^ CFU/mL, respectively, in the first soil sample, and they were higher than those for GA and PVA (Table 5). After 30 and 60 days, pure GA (100%) had a higher CFU than pure PVA (100%). The CFU values measured after 30 and 60 days for each of the six bioplastic sheets showed no discernible differences.

**Figure 15:**
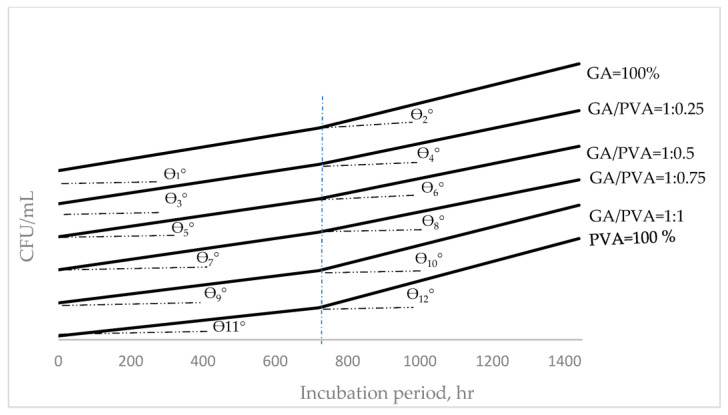
Hourly duplication (HD) in colony-forming units (CFUs) of the bacterial population in the buried NDBs blended from gum Arabic (GA) and polyvinyl alcohol (PVA) in different ratios.

**Figure 16:**
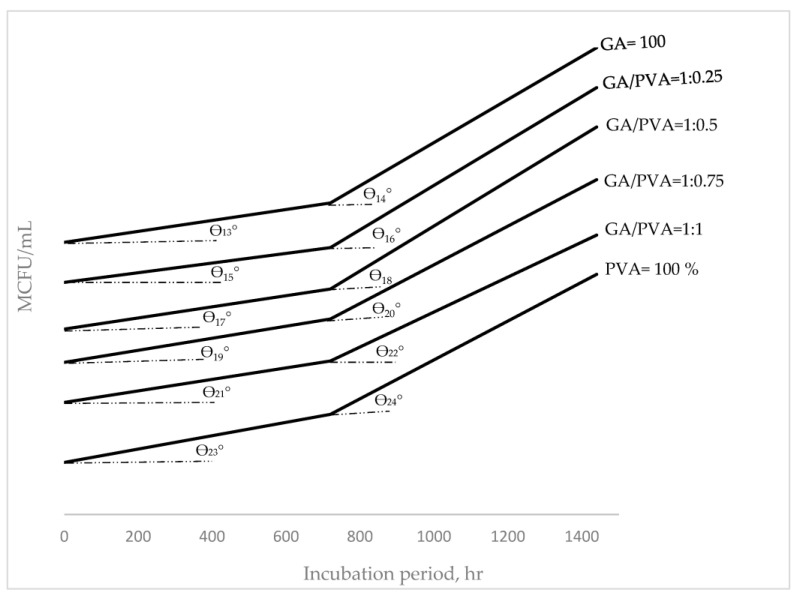
Hourly duplication (HD) in colony-forming units (CFUs) of fungi populations in the buried NDBs blended from gum Arabic (GA) and polyvinyl alcohol (PVA) in different ratios.

In addition, increasing colony-forming units (CFUs) over a defined period measured in hours was termed as hourly duplication (HD) of the CFU and was presented in Figure 15 and Figure 16 for bacteria and fungi, respectively. For the prediction of the HD’s values within the determined incubation periods, functional formulas were mathematically derived to reach this target, which is presented in Table 7.

Belonging to both bacterial (Figure 15) and fungi (Figure 16) communities, the HD values determined during and just after 800 h and up to 1400 h for each of the six bioplastic sheets showed the same ascending trend. For both trends, the HD’s mean values for all the blends’ ratios through the 1st duration (0–800 hrs.) showed a slower duplication rate than those within the 2nd region (800–1400 h). These duplication rates can be noticed from the slop angle of the HD curves, as shown in Figure 15 and Figure 16 [117,118].

In addition, it can also be seen when comparing the HDs of bacteria (Figure 15) with those for fungi (Figure 16) that the HDs’ rate for fungi communities grown on the six bioplastic membranes was higher than those recorded for their analogous curves belonging to the bacteria. Moreover, the higher level of HD for the fungi community was more obvious at the 2nd stage of the incubation period. This finding can be observed by speculating the curves’ slopes (tan θ_n_, where n = 1–24).

## 4. Discussion

### 4.1. Scientific Illustration of the Ease of Peeling the Bioplastic Membranes Away from the Acrylic Platform

In addition to the issue of drying the bioplastic sheets facing all hydrophilic natural polymer-based membranes, peeling these sheets to be rolled up is a major problem in the hydrophilic bioplastic blend field. Studying the ease of peeling the bioplastic membrane away from the casting panel template was achieved by investigating the chemical and physical properties of each of three parameters, namely, the bioplastic blend (fluid phase), the PMMA’s platform (solid phase), and the liquid/solid interface, as shown in Figure S4 [57,98,119,120,121,122,123,124,125].

Regarding the triboelectric series that classifies materials based on their propensity to take electrons (tribo-positive) or not (tribo-negative), it is important to note that PMMA is a biocompatible polymer, which due to its propensity for either donating or absorbing electrons, occupies the middle position on the triboelectric series [119].

The following examples show how simple it is to separate the bioplastic membrane from the acrylic platform:

- Materials with relatively low surface energies are regarded as non-stick surfaces [125] and vice versa. As shown in Table S1, the acrylic platform exhibits modest surface energy (41 dynes/cm) and contact angle (82°), both of which are indicative of a non-stick surface [126].
- Acrylic is a powerful static generator in terms of electrostatic charge. When its surface is wiped back and forth, positive and negative surficial charges arise that draw and hold microscopic particles. Surficial charge variations have the potential to cause agglomerated particles to discharge in an unanticipated manner, endangering contamination-sensitive materials [127]. PMMA is positioned close to the middle of this empirical series for the surface potential and is regarded as a tribo-positive electron-donating material [119,127].

GA is composed of three distinct fractions, as shown in Figure S3, including the arabinogalactan–protein complex (MW 1500 kDa; approximately 10% of the total gum solids), arabinogalactan (MW 280 kDa; approximately 88% of the total gum solids), and glycoprotein (MW 250 kDa; approximately 2% of the total gum solids [128,129,130]. Due to their low molecular weight and branching pattern, arabinogalactan films are challenging to produce [131,132].

According to Winiewska et al. [133], PVA chains have a particular percentage of acetate groups (14%), which are the source of the polymer molecules negative charges. The structure of the PVA adsorption layer is impacted by even the comparatively modest portion of these groups. The presence of more acetate groups in the polymeric chains resulted in increased PVA adsorption levels, indicating that these groups are crucial to PVA adsorption [134]. As the pH of the solution rises, so does the contribution of charged acetate groups.

Due to the electrostatic attraction of negative charges present along the polymeric chains, the polymer chains extend further. The amount of PVA is directly influenced by the degree of development in the polymer macromolecules.

In general, plastics are categorized into four categories by Nuraje et al. [135]: super hydrophilic, hydrophilic, hydrophobic, and super hydrophobic, with contact angles (θ°) of below 5°, below 90°, 90°–150°, and 150°–180°, respectively.

The liquid–fluid–solid system exhibits three different interfaces in its configuration when a liquid drop is placed on a solid surface (Figure S4), namely, liquid–fluid, solid–fluid, and liquid–solid. It is noticed that adhesive and cohesive forces are present at each interface as a result of the intermolecular forces at work there. Cohesive forces cause the drop to return to its spherical shape, whereas adhesion forces encourage it to spread out. The conflict between these two forces determines the contact angle [57,73,133,134,135,136,137]. It is feasible to establish a connection between the static contact angle and the interfacial stresses under equilibrium conditions. The Young–Dupre equation is the name of this relationship. By applying the Hild [126] formula, it was discovered that the spreading of a droplet of a bioplastic blend is equal to A − (B + C), where A is the surface tension of the bioplastic blend, B is the surface tension of the acrylic panel, and C is the surface energy of the interface between the bioplastic blend and the acrylic panel. While liquid will spread when the spreading is zero to positive, it will not if the spreading is negative.

### 4.2. Scientific Illustration of the Nanodehydration of the Bioplastic Membranes

It is well known that drying bioplastic membranes is a critical issue when manufacturing these products. This crucial problem arises from the highly hydrophilic nature of their natural-based precursors, such as the hydrophilic GA and PVA used in this study. Accordingly, a novel technique and device were invented to accelerate the dehydration process of such products [57]. The invention, termed the stratified nano-dehydrator (SND), is constructed from accurately selected materials, including perforated acrylic panels (poly-(methyl methacrylate)), polypropylene’s non-woven textile, cellulosic cotton floss, and an effective dehydrant agent such as P2O5, which is the most effective dehydrant reagent, rather than calcium chloride, magnesium sulfate, aluminum oxide, lithium aluminum hydride, metallic sodium, or silica gel.

The reasons for choosing PMMA material as barrier panels within the dehydrator apparatus were due to its self-electrostatic charging property as well as its ability to force the evaporated water molecules to have higher surface tension, which facilitates their escape outside the SND atmosphere and, subsequently, accelerates drying the bioplastic membranes. Furthermore, the cellulosic material (loosened Egyptian cotton floss) was selected for this task due to its high content of alpha cellulose, well-known for its high hydrophilicity, which is essential to attaining good affinity to both moisture molecules as well as dehydrant crystals.

The scientific concepts of ion dehydration reported by Pavluchkov et al. [138] can be used to explain the water molecules’ diffusion, especially in the transition state, by converting the water liquid into gaseous or steam matter.

Polar covalent bonds between the O- and H-atoms were extensively reported to generate an asymmetric distribution of electrons in a water molecule, with two excess electrons on the O-atom’s side of the molecule and two deficient electrons on the H-atom’s side. This asymmetry accepts water molecules with both molecular cohesion that attracts water molecules to each other and/or adhesion that attracts water molecules to their neighboring asymmetrically charged materials like ions, polarized molecules, and charged surfaces, including but not limited to glass [139] and the PMMA’s casting platform used in the present investigation [57].

Since water molecules have high polarity, their transportation (evaporation) from the bioplastic membranes upon the drying process can be viewed like the ion dehydration phenomenon that regulates ionic transport through sub-nanopores, which can permit selectivity between similar sized and charged ions, as referred to by Pavluchkov et al. [138]. Transition-state theory gives an idea about molecular activation parameters (enthalpy and entropy) that determine the interaction level between the transported species and the wet bioplastic blend, as well as the freedom of molecular motion within it. Since hydration and dehydration effects are characterized by substantial enthalpic (due to changes in the chemical bonds between the ion and its surroundings) and entropic (due to changes in the spatial structure of the ion) changes at the molecular level, this theory has been successfully suggested to explore dehydration-related transport phenomena in membranes [138]. 

The enclosed system within the SND was partially vacuumed to give mildly driven forces that accelerate the water vapor molecules’ escape outside the dehydrator [57] and subsequently enhance the drying process itself.

### 4.3. Chemical and Physical Properties of the Bioplastic Membranes

#### 4.3.1. FTIR

Different organic functional groups found in naturally occurring substances can be recognized using Fourier transform infrared (FTIR) spectroscopy. The complicated vibrational modes were seen in the FTIR spectra for the various bioplastic samples over a wide range of wavenumbers (Figure 4).

Figure 4 shows the strong and broad O-H stretching vibrations at 3416 cm^−1^ dominating the primary FTIR spectra of the six bioplastic sheets. At 2939 cm^−1^, the C-H stretching modes are riding above the board peak. Along with the bulk ring mode at 1426 cm^−1^, the carbonyl stretching modes are seen at 1641 cm^−1^. At 1047 cm^−1^, the typical C–O–C anti-symmetric stretching mode was found. These findings are modified from those attained by other researchers [108,109,110,111] for the study of biopolymeric materials.

For an additional illustration, the overall banding of the FTIR analysis showed a carbohydrate fingerprint at 900–1250 cm^−1^ [138]; C–O–C anti-symmetric stretching at 1426 and 1047 cm^−1^ [57,98,99]; COO^−^ asymmetric stretching at 1402 cm^−1^ [139]; an O–H in-plane bending band in carboxylic acids at 1625.4, 1627.4, 1430, 1436.91, and 1437 cm^−1^ [132,133]; COO^–^ symmetric stretching and carbonyl stretching modes at 1641 cm^−1^ [57,98,99,101]; C–H stretching at 2800–3000, 2885, and 2939 cm^−1^ [57,98,99,101,137,140,141,142]; vibrational modes of the C–H group at 2910.87 cm^−1^ [141,142]; O–H stretching vibrations at 3261, 3416, and 3000–3600 cm^−1^ [57,98,137,141,143], and the unique presence of O–H groups at 3526.35 cm^−1^ [142,143].

The FTIR for the NDB used in the current work and the ADB created by Hindi et al. [98] have main functional groups that share chemical characteristics, according to the comparison. As a result, the chemical components of the bioplastic products have been preserved by the use of innovative casting blends, nano-dehydration, and membrane peeling.

#### 4.3.2. XRD

The GA-broad diffractogram’s greatest intensity was recorded at 2 θ° = 20° (Figure 5), which supports the amorphous nature of gum Arabic [13]. Moreover, a typical peak for pure PVA, a semi-crystalline polymer detected at 2 θ° = 19.9° (Figure 4f), confirmed its semi-crystallinity feature [57,92,111].

With the increase in PVA allocation in the blend, the crystallinity index values increased. The growing CI of the bioplastic blends can be correlated to the increasing PVA allocation in the blend because the CI value of PVA (54.81%) was found to be greater than that of GA (19.4%).

#### 4.3.3. TGA

TGA analyzes the mass change behavior in bioplastic membranes that occurs as a function of temperature and time in a controlled environment. The best uses for it are to evaluate reaction kinetics, volatile contents, thermal stability, degradation traits, aging/lifetime breakdown, and degradation features.

The thermal deterioration of the samples (Figure 6) increased at higher temperatures (up to 500 °C) than at lower temperatures, according to a comparison of the mass losses between the temperature zones. Furthermore, a comparison of the mass losses across the temperature ranges revealed that PVA shed more weight in the higher temperature zones than GA. A mass loss of up to 100 °C can be attributed to the water molecule’s large solvation capacity, which results in the evaporation of loosely bound moisture on the surface, or “free water”. Furthermore, mass loss at temperatures up to 150 °C can be due to hygroscopic water evaporation [57,92].

#### 4.3.4. DTA

Similar information is provided by DTA, which measures the temperature difference between a sample and a reference due to thermal treatments in a material. The DTA typically provides phase transition information in addition to the TGA.

It is commonly known that two types of thermograms can be distinguished for a given material during thermal reactions: endothermic, which uses energy, and exothermic, which excludes energy. The depolymerization of the bioplastic materials themselves as a result of heat treatment causes exograms to occur (Figure 7). Moreover, the endotherm can be attributed to the fusing or melting of crystallites as well as the evaporation of free moisture (up to 100 °C) and hygroscopic moisture (up to 120 °C) [57].

As shown in Figure 7 and Table 3, GA had the lowest value of heat change (−1017.3 Vs/mg), but the endotherm of pure PVA absorbed the maximum amount of energy (2119.7 Vs/mg) among the other bioplastic blends. As a result, PVA is more thermally stable than GA because it absorbs heat more effectively, shielding the bioplastic sample from potential thermal degradation brought on by rising temperatures. Moreover, the enhanced PVA allocation in the blends boosted the thermal stability of the bioplastic sheets.

GA exhibits greater thermal stability than PVA at higher temperatures (about 350 °C). As a result, altering a bioplastic blend to increase PVA or decrease GA enhances the thermal stability of the resulting bioplastic membrane [57,92].

### 4.4. Anatomical Ultrastructure of the Bioplastic Membranes

#### 4.4.1. Surface Roughness and Nanometric Particle Size

While the pure PVA sheets (0/1 blend ratio) had the greatest PS values for both the ADB and NDM, the pure GA membrane (1/0) had the lowest PS values. Gradually raising the PVA concentration in the bioplastic blend increased the PS. The surface roughness (SR) features examined via atomic force microscopy (AFM), as shown in Table 4 and Figure 8, provide confirmation of this. The results obtained for PVA-based membranes and the median value for those cast from GA/PVA (1:1) are compared. 

As a result, the presence of PVA causes the SR of the water-based polymeric blends to increase, generating a surface that is rougher. This is supported by the surface roughness features examined using atomic force microscopy (AFM), as shown in Table 4 and Figure 8.

Increases in PVA allocation make the blend’s texture coarser since PVA membranes have a rougher structure than GA membranes. According to the comparison of the membranes, there is no statistically significant difference between the ADB and NDB in their PS, along with the six bioplastic blends. Additionally, analyses of the effects of different bioplastic blend ratios on a membrane showed that, in both cases, blends with higher PVA concentrations produced membranes with higher porosities (PD and VV).

It is important to note that smoother sheets are preferred for packaging over coarser ones because the latter tend to gather more dust on their surfaces. Although PVA is a crucial part of the bioplastic mixture that improves the quality of the final membrane and makes it easier for it to peel off the casting surface after drying, a careful balance must be taken into account to have the best quality and smoothest surfaces.

Moreover, there is no statistical distinction in the PS between the ADB and the NDB. Because of this, the unique techniques created to make it easier to cast their mixes, dry them faster, and peel membranes off easily using a self-electrostatic template did not alter the parent roughness properties. Because of this, the unique approaches employed in the current study did not alter the permeability of the membranes (PD and VV).

#### 4.4.2. Membrane Permeability

For the membrane ultrastructure presented in Figure 9, the GA membranes had the lowest PD and VV compared to those for PVA, which had the highest ones for both air-dried bioplastic (ADB) membrane and nanodehydrated transparent bioplastic (NDB) membrane. Accordingly, increasing the PVA allocation increased the membrane permeability, which facilitated the water evaporation from the blend during the nano-dehydration procedures.

When compared to PVA membranes, which had the highest PD and VV for both the air-dried bioplastic (ADB) and nanodehydrated (NDB) membranes, the GA membranes had the lowest ultrastructures. As a result, increasing the PVA allocation also increased the membrane permeability, which made it easier for the blend’s water to evaporate throughout the nano-dehydration processes.

Moreover, there is no statistical difference between the ADB and NDB for each case of the PS, PD, or VV. Because of this, the unique approaches employed in the current study did not alter the permeability of the membranes (PD and VV), as shown in Figure 9 and Table 4.

### 4.5. Mechanical Properties of the Bioplastic Membranes

The evaluation of a film’s capability and mechanical integrity heavily relies on its mechanical properties. The interactions between the blend’s components had a significant impact on the matrices of blended films. The mechanical properties were reported to be solely dependent on the chemical structure, which could be best described by using UTS, MoE, and EaF [74]. In addition, it was reported by Gomaa et al. [77] that the internal molecular force, the crystallinity shape, and the content of the polymer all have a significant impact on the mechanical characteristics [77].

The findings understood from Table 5 and Figure 11 revealed that both ADB (the red curve) and NDB (the blue curve) are similar in their ascending trend starting from the PL level up to the US. This behavior means that each membrane, else ADB or NDB was stressed through two stages: (1) in the 1st one, the stress was increased from zero up to the PL level, (2) through the 2nd stage, each membrane transitioned from elastic to plastic nature as the load was increased from the PL up to the maximum load resulting the US stage.

In addition, regarding to proportionality limit (PL) of the bioplastic membranes, it is higher for the NDB than that for the ADB for all the six blend ratios. This indicates that the NDB membranes has higher elasticity endurance compared to their analogous membranes.

As clear from Table 5 and Figure 11, the plotted stress-strain curves for the six blended membranes were differed concerning to their proportionality limit (PL) and ultimate strength (US).

Regarding to sub-graphs of the bioplastic membranes in Figure 11a–f, the similarity between the ADB and NDB in their ascending trend starting from the PL level up to the US can be explained that these membranes was stressed through two stages: (1) in the 1st one, the stress was increased from zero up to the PL level, and (2) through the 2nd stage, each membrane transitioned from elastic to plastic nature as the load was increased from the PL up to the maximum load resulting the US stage.

Since the proportionality limit (PL) of the bioplastic membranes was found to be higher for the NDB than that for the ADB for all the six blend ratios. This indicates that the NDB membranes has higher elasticity endurance compared to their analogous membranes.

The highest values of the UTS (Figure 12) and the MoE (Figure 13) for the membranous sample at the blend ratio of 1:0.25 can be attributed to the strong interaction between the GA and PVA at this optimum blend ratio, which permitted complete miscible blending [76].

The EaF of the bioplastic film samples is explained by the maximum change in its length before failure or breaking as clear from Figure 14 [74].

As shown in Figure 14, adding the GA to the blends enhanced the EaF’s membranes up to the blend ratio of GA/PVA of 0.5/0.5. This could be attributed to the good interfacial adhesion among the polymer components (GA and PVA). These findings of the mechanical study confirm the addition of gum acacia can improve mechanical properties, which decrease with an increase in the allocation of gum Arabic [74].

Adding polyvinyl alcohol to the gum Arabic for preparing the bioplastic blend films improved the mechanical properties of these membranes, especially in the blend ratio of GA/PVA of 1:0.25, which enhanced both UTS and MoE, while EaF was enhanced for the blend ratio’s membrane GA/PVA of 1:1. Therefore, the results of this work may show that the functional properties of GA/PVA blend films are adequate for food packaging applications and in the pharmaceutical industry for controlled release of drugs [74].

### 4.6. Microbial Biodegradation

Biodegradation of the NDB material was confirmed significantly by its reduction in weight for all six NDB samples, and it was found that degradation commenced within 30 and 60 days [144,145,146,147].

The microbial communities in all the buried bioplastic sheets, including the control one, were different in number and species. The species of bacteria and fungi differed according to the type of buried sheet.

The microbiological study revealed that all six bioplastic sheets are able to be degraded, contrary to petroleum-based sheets.

Biodegradation is the process by which microorganisms can degrade bioplastic membrane materials, leading to a loss of weight after a period of time. Our results show that all blended bioplastic membranes have reduced weight, especially GA. Our results agree with those of Sasaki et al. [92], who prepared films of phenolic extracts incorporated into GA and found that the highest weight loss of films was 45.81%, compared with GA (26.87%) after 30 days.

Microorganisms can degrade bioplastic membranes through a process called biodegradation, which eventually causes the membranes to lose their weight. Our findings indicate that the weights of all blended bioplastic membranes, particularly GA, decreased. Our findings are consistent with those of Sasaki et al. [92], who created films using phenolic extracts mixed with GA and discovered that after 30 days, the weight loss of the films was higher than that of the GA (45.81%).

These findings were contrary to Ibrahim et al. [78], who discovered that for nanofiber membranes based on homogenous polymeric blends of gum Arabic, polyvinyl alcohol, and silver nanoparticles, the biodegradation tests of the generated nanofibers revealed that 99.09% of the material was broken down after 28 days (Table 7). These variations in the results can be explained by the fact that a variety of factors, including microbes, humidity, sunshine, and oxygen, can affect the bioplastic’s capacity to degrade [80].

In addition, because it affects the microbial population and shapes it, the depth of the soil that bioplastic membranes are buried in is a crucial component for biodegradation [78]. In addition, as a result of using gum as a source of nutrients, the number of bacteria increased over time [82,98].

Our results proved that *Pseudomonas* spp., *Bacillus* spp., and *Micrococcus* spp. were the most commonly isolated bacterial strains appearing in different samples, while *Rhizobus* spp., *Penicillium* spp., and *Fusarium* spp. were the most commonly isolated fungus strains that appeared in our different samples. These findings agree with those found by Santos–Beneit et al. [93] and Sasaki et al. [92], which were isolates of *Bacillus cereus*, *Bacillus polymyxa*, *Bacillus licheniformis*, *Corynebacterium xerosis*, *Staphylococcus epidermis*, *Streptococcus bovis*, and the fungi *Penicillium notatum*, *Rhizopus nigricans*, *Aspergillus niger*, and *Fusarium moniliforme* from gum Arabic [68,87,89,90,92,144,145].

Belonging to comparisons within communities, it was found that the HD values determined during and just after 800 h and 1400 h for each of the six bioplastic sheets buried in the soil were similar in their trend concerning each of the bacteria (Figure 15) and fungi (Figure 16) as well as Table 7. This similarity in trends can be attributed to the constancy of the burying depth of the bioplastic membranes [78] and/or various factors, including microbes, humidity, sunshine, and oxygen, which can affect the bioplastic’s capacity to degrade [80].

Moreover, a common trend was registered between the NDB products fabricated in the current investigation and the ADB synthesized by Hindi et al. [98] and Hindi and Albureikan [57]. Accordingly, the nano-dehydration invention did not affect the parent’s ability to biodegrade the bioplastic membranous product.

## 5. Conclusions and Future Perspectives

Great success was achieved for the fabrication of bioplastic membranes from gum Arabic mixed with polyvinyl alcohol by applying a novel casting method, termed static vibrated-free horizontal flow, which produces free air bubble sheets. The novel nano-dehydration technique gave the best solution for drying the bioplastic sheets and can be used for any water-based biopolymeric-based product. It is the first time that an acrylic (poly-(methyl methacrylate)) panel used as an ideal template surface features an electrostatically charged hydrophobic surface. As a result, peeling off its template surface is made simpler.

The most important properties of the nanodehydrated bioplastic membranes were studied using Fourier transform infrared spectroscopy, X-ray powder diffraction, thermogravimetric analysis, differential thermal analysis, and atomic force microscopy to ensure that the novel techniques did not distort the product quality. The nanodehydrated bioplastic membranes retained their parent properties, including chemical functional groups, crystallinity index, mass loss, thermal stability, ultrastructure features (surface roughness and permeability), and their ability for microbial biodegradation. PVA had a higher crystallinity index (CI), a greater mass loss at higher temperatures, higher thermal stability due to its higher heat content, and greater clearance of surface roughness due to its high particle size (PS), as well as higher permeability parameters, namely, pore diameter (PD) and void volume (VV), than those for GA. Accordingly, increasing the PVA allocation in the bioplastic blends could enhance their properties except for mass loss, whereas increasing the GA allocation in the NDB blend reduced its mass loss at elevated temperatures. 

There is no statistical difference between the bioplastic membranes synthesized elsewhere with ordinary air drying or nano-dehydration in terms of their particle size and permeability, indicating that the novel procedures used did not distort the parent properties examined as well as their ability for biodegradation. Adding polyvinyl alcohol to the gum Arabic for preparing the bioplastic blend films improved the mechanical properties of these membranes, especially in the blend ratio of GA/PVA of 1:0.25, which enhanced both UTS and MoE, while EaF was enhanced for the blend ratio’s membrane GA/PVA of 1:1. Therefore, the results of this work may show that the functional properties of GA/PVA blend films are adequate for food packaging applications and in the pharmaceutical industry for controlled release of drugs [74]. The biodegradation of the nanodehydrated bioplastic membranes was confirmed significantly by the reduction in weight for all six blended samples, and degradation was found to start within 30 and 60 days. Pure GA was the most commonly biodegraded sample among the other bioplastic samples. The microbial communities in all of the buried bioplastic sheets, including the control sample, were different in number, species, and duplication rates. The microbiological survey revealed that all six bioplastic sheets are able to be degraded, contrary to petroleum-based sheets.

## 6. Patent

System, apparatus, and methods for manufacturing biodegradable biopolymeric materials (US Patent No. 11548192).

## Figures and Tables

**Table 1:** Arithmetic formulas for calculating different chemical and physical properties of the bioplastic sheets.

Equation	Definitions
^1^ CI = (A_pp_/A_t_) × 100	A_pp_: Total planar area (mm^2^) of the XRD’s diffractogram.
	A_t_: The planar area (mm^2^) of the principle peaks arises around 2θ° of 20°.
^2^ M_L_ = [(W_2_ − W_1_)/W_1_] × 100	W_i_: Initial weight of the bioplastic sample at a temperature zone, estimated from the TGA curves. W_f_: Final weight of the bioplastic sample at the same temperature zone, estimated from the TGA curves.
^3^ TR = T_i_ − T_f_	T_i_: Initial temperature of a certain thermogram. T_f_: Final temperature of the same thermogram.
^4^ EC = E_f_ − E_i_	E_f_: Initial enthalpy of a certain thermogram. E_i_: Final enthalpy of the same thermogram.
^5^ TS = F_f_/A ^6^ MoE = σ/ε ^7^ ε = [∆L/L_o_] = [(L_f_ − L_○_)/L_○_] ^8^ EaF = ∆L_f_ = [(L_f_ − L_○_)/L_○_] × 100	F_f_: Force at failure in Newton (N).
	A: Cross-section area (m^2^) of the bioplastic sample.
	σ: Tensile stress (Pa).
	L_f_: The length of the bioplastic sample at failure.
	L_○_: The initial length of the bioplastic sample at failure.
^9^ Tan (θ_n_°) = (y_2_ − y_1_)/(x_2_ − x_1_) y = mx + C m = Tan (θ_n_°)	θ°: The incline angle (θ) in degrees of the hourly duplication (HD) curve for microbial populations. x_1_: The 1st incubation period (hr.) for the HD curve. x_2_: The 2nd incubation period (hr.) for the HD curve. y_1_: The initial HD of the colony-forming units (CFUs) in mega units (MCFU). y_2_: The final HD of the colony-forming units (CFUs) in mega units (MCFU). m: The slope of the curve C: The intersecting section of the y-coordinate for the HD’s curve.

^1^ Crystallinity index (%),^2^ Mass loss of the bioplastic membrane (g), ^3^ Temperature range (°C), ^4^ Enthalpy change (µV/mg), ^5^ Tensile strength (MPa), ^6^ Modulus of elasticity (GPa), ^7^ Tensile strain, ^8^ Elongation at failure (%), ^9^ Slope of the HD-curve.

**Table 2:** Mean ^1–7^ values of mass loss (ML) of the six transparent nanodehydrated bioplastic (NDB) membranes over a wavenumber range of 4000 to 500 cm^−^^1^, fabricated from various gum Arabic (GA)/polyvinyl alcohol (PVA) blends over different ratios and temperature (T) zones.

T-Zones °C	GA/PVA Ratio					
	GA 100%	1:0.25	1:0.5	1:0.75	1:1	PVA 100%
50°–100°	13.68 ^A^_d_	4.57 ^B^_g_	4.85 ^B^_e_	5.02 ^B^_ef_	5.23 ^B^_ef_	5.69 ^B^_f_
100°–150°	11.11 ^A^_e_	6.32 ^AB^_f_	6.37 ^AB^_e_	6.93 ^AB^_e_	6.47 ^AB^_ef_	8.98 ^B^_ef_
150°–200°	4.18 ^BC^_g_	4.88 ^BC^_g_	6.46 ^B^_e_	4.26 ^BC^_ef_	3.23 ^C^_f_	8.07 ^A^_ef_
00°–250°	6.96 ^B^_f_	8.18 ^A^_e_	6.18 ^B^_e_	3.7 ^C^_f_	5.71 ^B^_ef_	3.9 ^C^_g_
250°–300°	7.48 ^C^_f_	14.9 ^A^_d_	7.36 ^C^_e_	10.77 ^AB^_d_	11.62 ^B^_d_	10.15 ^AB^_e_
300°–350°	22.2 ^B^_b_	12.62 ^E^_de_	15.93 ^D^_d_	22.41 ^B^_c_	28.57 ^A^_c_	18.08 ^C^_d_
350°–400°	18.2 ^E^_b_	21.13 ^D^_b_	26.32 ^C^_b_	33.3 ^B^_b_	34.4 ^B^_b_	44.14 ^A^_c_
400°–450°	28.6 ^D^_a_	27.68 ^D^_a_	41.43 ^C^_a_	39.17 ^BC^_a_	46.34 ^B^_a_	79.0 ^A^_a_
450°–500°	15.6 ^E^_c_	18.52 ^D^_c_	21.95 ^CD^_c_	24.66 ^C^_c_	45.5 ^B^_a_	58.8 ^A^_b_

^1^ Each value is an average of 3 samples. ^2^ Based on the original oven-dry weight. ^3^ Superscript capital letters for comparing blend ratios within the same temperature zone. ^4^ Subscript small letters for comparing temperature zones within the same blend ratio. ^5^ Means with the same letter are not significantly different at the 5% level. ^6^ Initial starting weight of the NDBs sample. ^7^ Final starting weight of the NDB sample.

**Table 3:** DTA results of the six transparent nanodehydrated bioplastic (NDB) membranes in the wavenumber range of 4000 to 500 cm^−^^1^, fabricated from various gum Arabic (GA)/polyvinyl alcohol (PVA) blends: points of reaction, thermogram type, temperature range (TR), and enthalpy change (EC).

Points of Reaction	GA/PVA Ratio	Thermogram Type	TR °C	EC µVs/mg
a	GA = 100%	Endotherm Exotherm	25–265 265–435	−1017.25 +52.39
b	1:0.25	Endotherm	25–475	−2268.77
c	1.0.5	Endotherm Exotherm	25–397 397–480	−1127.7 −16.67
d	1:0.75	Endotherm Exotherm	25–375 375–475	−1276.04 −20.89
e	1:1	Endotherm	25–475	−1467.19
f	PVA = 100%	Endotherm	25–475	−2119.72

**Table 4:** Statistical parameters (SPs) of the ultrastructural features of the bioplastic membranes.

GA/PVA Ratio	GA Amount %	PVA Amount %	SPs	Particle Size nm		Permeability			
						Pore Diameter nm		Void Volume nm^3^	
				ADB	NDB	ADB	NDB	ADB	NDB
1/0	0	100	Mean ^1,2^	13.57	14.77	0.91	0.953	83.24	84.29
			Max. ^3^	55.44	56.68	3.905	3.948	1397.9	1398.91
			Min. ^4^	4.24	5.49	0.002	0.045	0.007	1.057
			SD ^5^	7.66	7.66	0.904	0.904	160.68	160.68
1:0.25	20	80	Mean ^1,2^	14.17	15.42	0.553	0.606	105.74	106.74
			Max. ^2,3^	76.94	78.19	3.54	3.593	1374.8	1375.47
			Min. ^2,4^	4.24	5.49	0.001	0.055	0.005	1.008
			SD	8.93	8.93	0.457	0.457	156.17	156.17
1:0.5	66.7	33.3	Mean ^1,2^	15.15	16.4	0.608	0.671	120.66	121.87
			Max. ^2,3^	67.01	68.26	2.38	2.443	8009	8010.22
			Min. ^2,4^	4.24	5.49	0.001	0.064	0.002	1.219
			SD ^5^	8.51	8.51	0.469	0.469	309.6	309.6
1:0.75	57.1	42.9	Mean ^1,2^	17.01	18.07	0.714	0.788	226.98	228.18
			Max. ^2,3^	72.32	73.38	3.608	3.683	8411.8	8412.98
			Min. ^2,4^	4.24	5.3	0.007	0.082	0.007	1.215
			SD ^5^	9.26	9.26	0.615	0.615	631.41	631.41
1:1	50	50	Mean ^1,2^	18.42	19.58	1.145	1.23	460.18	461.5
			Max. ^2,3^	89.75	90.91	4.75	4.839	8411.8	8413.1
			Min. ^2,4^	4.24	5.4	0.019	0.093	0.007	1.34
			SD ^5^	12.69	12.69	2.342	1.002	1062.04	1062.04
0/1	100	0	Mean ^1,2^	20.34	21.35	1.485	1.58	548.95	552.41
			Max. ^2,3^	89.75	90.76	14.851	14.946	9315	9318.46
			Min. ^2,4^	4.24	5.25	0.019	0.114	0.001	3.46
			SD ^5^	14.58	14.58	2.342	2.342	1198.36	1198.36

^1^ Mean of the population members. ^2^ The number of observations is 1000 individuals. ^3^ Max. is the maximum value. ^4^ Min. is the minimum value. ^5^ SD are standard deviation values present within the parentheses.

**Table 5:** Proportionality limit (PL) and ultimate stress (US) of the six blends for each of the air-dried (ADB) and nanodehydrated (NDB) membranes.

GA/PVA Ratio	GA Amount %	PVA Amount %	Stress Type	ADB		NDB	
				Stress MPa	Strain	Stress MPa	Strain
1/0	0	100	PL	8.1	29.69	11.34	37.42
			US	20.42	145.24	21.42	145.25
1:0.25	20	80	PL	14.24	41.22	17.61	39.73
			US	28.91	319.13	31.05	300.9
1:0.5	66.7	33.3	PL	8.92	46.47	9.56	50.56
			US	18.84	249.5	20.64	264.98
1:0.75	57.1	42.9	PL	7.15	48.4	10.04	56.3
			US	15.92	269.39	19.7	284.39
1:1	50	50	PL	4.92	38.41	4.92	38.41
			US	11.86	297.99	12.33	310.55
0/1	100	0	PL	12.03	65.26	15.3	72.61
			US	21.64	227.09	25.47	264.91

**Table 6:** Colony-forming units (CFUs) of microbial populations for bacterial and fungal species in the buried NDBs blended from gum Arabic (GA) and polyvinyl alcohol (PVA) in different ratios for soil burying.

AG/PVA Ratio	After 30 Days		After 60 Days	
	Bacteria CFU/mL	Fungi CFU/mL	Bacteria CFU/mL	Fungi CFU/mL
GA = 100%	2.8 × 10^6^ ^1^ [0.032]	1.77 × 10^3^ [0.008]	6.69 × 10^6^ [0.086]	4.32 × 10^3^ [0.077]
1:0.25	2.6 × 10^6^ [0.07]	1.8 × 10^3^ [0.042]	5.86 × 10^6^ [0.074]	3.8 × 10^3^ [0.093]
1:0.5	2.52 × 10^6^ [0.028]	1.88 × 10^3^ [0.094]	5.7 × 10^6^ [0.064]	4.21 × 10^3^ [0.086]
1:0.75	2.5 × 10^6^ [0.031]	1.93 × 10^3^ [0.095]	5.67 × 10^6^ [0.095]	4.02 × 10^3^ [0.086]
1:1	2.17 × 10^6^ [0.088]	1.9 × 10^3^ [0.012]	6.14 × 10^6^ [0.088]	3.79 × 10^3^ [0.044]
PVA = 100%	1.93 × 10^6^ [0.008]	2.1 × 10^3^ [0.083]	6.12 × 10^6^ [0.093]	4.83 × 10^3^ [0.046]
Soil control sample	Bacteria: 2.28 × 10^5^ CFU/mL [0.058]			
	Fungi: 1.28 × 10^3^ CFU/mL [0.022]			

^1^ Values within parentheses are standard deviations.

**Table 7:** Functional relationships between the incubation period (IP) as an independent variable (x) and hourly duplication (HD) as a dependent variable (y) of bacteria and fungi populations in the buried NDBs blended from gum Arabic (GA) and polyvinyl alcohol (PVA) in different ratios.

Microbial Type	AG/PVA Ratio	HD-Equation			
		^1^ IA	The 1st Stage (0–720 h)	IAS	The 2nd Stage (720–1400 h)
Bacteria	GA = 100%	θ_1_°	y = 3572.2x + 228 × 10^3^	θ_2_°	y = 8975x + 228 × 10^3^
	1:0.25	θ_3_°	y = 3294.4x + 228 × 10^3^	θ_4_°	y = 7822.2x + 228 × 10^3^
	1:0.5	θ_5_°	y = 3183.3x + 228 × 10^3^	θ_6_°	y = 7600x + 228 × 10^3^
	1:0.75	θ_7_°	y = 3155.56x + 228 × 10^3^	θ_8_°	y = 7558.3x + 228 × 10^3^
	1:1	θ_9_°	y = 2697.2x + 228 × 10^3^	θ_10_°	y = 7100x + 228 × 10^3^
	PVA = 100%	θ_11_°	y = 2363.89x + 228 × 10^3^	θ_12_°	y = 6794.4x + 228 × 10^3^
Fungi	GA = 100%	θ_13_°	y = 0.8194x + 1.28 × 10^3^	θ_14_°	y = 3.2361x + 1.87 × 10^3^
	1:0.25	θ_15_°	y = 0.7222x + 1.28 × 10^3^	θ_16_°	y = 3.3333x + 1.8 × 10^3^
	1:0.5	θ_17_°	y = 0.8333x + 1.28 × 10^3^	θ_18_°	y = 3.375x + 1.88 × 10^3^
	1:0.75	θ_19_°	y = 0.9028x + 1.28 × 10^3^	θ_20_°	y = 2.90278x + 1.93 × 10^3^
	1:1	θ_21_°	y = 0.8611x + 1.48 × 10^3^	θ_22_°	y = 2.625x + 2.1 × 10^3^
	PVA = 100%	θ_23_°	y = x + 1.78 × 10^3^	θ_24_°	y = 2.9167x + 2.5 × 10^3^

^1^ Incline angle in degrees.

## Data Availability

The supporting data for the reported results, including a link to the publicly archived datasets analyzed or generated during this study, can be found under the following patent: US Patent for System, apparatus, and methods for manufacturing biodegradable biopolymeric materials (Patent #11548192, issued 10 January 2023)—Justia Patents Search, https://patents.justia.com/patent/11060208 (accessed on 17 November 2022).

## Supplementary Materials

The following supporting information can be downloaded at: https://www.mdpi.com/article/10.3390/polym15153303/s1, Figure S1. The practical procedure used for the novel casting of the (NDB) membranes; Figure S2. The vibrational casting process of the polymeric blends into sheets; Figure S3. Chemical constituents of the polymers used to synthesize the nanodehydrated-bioplastic (NDB) membranes: (a) gum Arabic (GA) precursor, (b) polyvinyl alcohol (PVA) precursor, (c) (poly-(methyl methacrylate), PMMA); Figure S4. Visualization analysis of void volumes (VV, nm^3^) of the six nanodehydrated-bioplastic membranes (NBMs): (a) GA (100%); (b) GA/PVA = 1:0.25; (c) GA/PVA = 1:0.5; (d), GA/PVA = 1:0.75; and (e) GA/PVA = 1:1, and (f) PVA = 100% based on AFM-image analysis; Table S1. Surface energy and contact angle of the most important industrial polymers; Table S2. Calculating means of mass loss (ML) of the NDB membranes blended from gum Arabic (GA) and polyvinyl alcohol (PVA) in the six ratios and different temperature zones (T-zones).

## Nomenclature

ADB	Air-dried bioplastic
ACS	The American Chemical Society
AFM	Atomic force microscopy
CI	Crystallinity index
CFU	Colony-forming unit of microbial populations
DSC	Differential scanning calorimetry
DTA	Differential thermal analysis
EaF	Elongation at failure
EC	Enthalpy change
FTIR	Fourier transform infrared spectroscopy
GA	Gum Arabic
HC	Heat change in µVs/mg
HD	Hourly duplication
MoE	Modulus of elasticity
µVs/mg	Microvolts per milligram
NDB	Nanodehydrated bioplastic		
NPS	Nanometric particle size
PubChem	Open chemistry database managed by the National Institutes of Health (NHI)
PVA	Polyvinyl alcohol
SD	Standard deviation
SECT	Self-electrostatic charged-template
SP	Statistical parameters
SR	Surface roughness
PD	Pore diameter
PS	Particle size
TGA	Thermogravimetric analysis
TR	Temperature range (°C)
UTS	Ultimate tensile strength
XRD	X-ray diffraction
VFHF	Vibrated-free horizontal flow
VV	Void volume
