# Supplementary material for: Fabrication, Characterization, and Microbial Biodegradation of Transparent Nanodehydrated Bioplastic (NDB) Membranes Using Novel Casting, Dehydration, and Peeling Techniques
Source: Polymers (Basel). 2023 Aug 4;15(15):3303. doi: 10.3390/polym15153303 (PMC10422282; doi:10.3390/polym15153303)
Supplement: Supplementary file 1 [file polymers-15-03303-s001.zip › polymers-2435333-supplementary.pdf]

Supplementary Materials

Fabrication, Characterization, and Microbial Biodegradation of Transparent Nanodehydrated Bioplastic (NDB) Membranes Using Novel Casting, Dehydration, and Peeling Techniques

Sherif S. Hindi [1,*] and Mona Othman I. Albureikan [2]

[1] Department of Agriculture, Faculty of Environmental Sciences, King Abdulaziz University (KAU),
P.O. Box 80208, Jeddah 21589, Saudi Arabia
[2] Department of Biological Sciences, Faculty of Science, King Abdulaziz University (KAU), P.O. Box 80208, Jeddah 21589, Saudi Arabia; malboraikan@kau.edu.sa
* Correspondence: shindi@kau.edu.sa

This PDF file includes:

Section S1. The casting technique steps.

Figure S1. The practical procedure used for the novel casting of the (NDB) membranes.

Section S2. The machinery of the novel casting method.

Figure S2. The vibrational casting process of the polymeric blends into sheets.

Section S3. Chemophysical features of the polymers used in the bioplastic fabrication.

Figure S3. Chemical constituents of the polymers used to synthesis of the nanodehydrated-bioplastic (NDB) membranes: a) gum Arabic (GA) precursor, b) polyvinyl alcohol (PVA) precursor, c) [poly(methyl methacrylate), PMMA].

Section S4. Wettability of different types of plastics.

Table S1. Surface energy and contact angle of the most important industrial polymers.

Section S5. Thermogravimetric Analysis (TGA) of the NDB membranes.

Table S2. Calculating means of mass loss (M_L) of the NDB membranes blended from gum Arabic (GA) and polyvinyl alcohol (PVA) in the six ratios and different temperature zones (T-zones).

Section S6. Estimation of pore diameter distribution.

Figure S4. Visualization analysis of void volumes (VV, nm^3) of the six nanodehydrated-bioplastic membranes (NBMs): a) GA (100%), b) GA/PVA=1:0.25, c) GA/PVA=1:0.5, d), e) GA/PVA=1:0.75, and f) GA/PVA=1:1, and (PVA=100%) based on AFM-image analysis.

Section S1. The casting technique steps

Mixing a pre-determined concentrations of GA and PVA via continuous stirring to obtain a homogenous blend solution.

Preparing and cleaning the casting panel and adjusting its slope angle to accelerate fluid movement of the blend solution.

Irradiation of the hydrophobic casting panel (Poly(methyl methacrylate) using UV-waves to: 1) activate its electrostatic charges, and 2) worming up the panel up to 50 ºC

Working vibrational forces on the casting panel.

Pouring the blind onto the vibrated hot casting panel in a definite velocity.

Applying a mild air drying of the molten polymeric membrane to thicken its viscosity.

Performing the accurate novel nano-dehydration technique using the novel stratified air-dryer apparatus.

Peeling off the bioplastic membrane from the casting panels and rolling it.

Figure S1. The practical procedure used for the novel casting of the bioplastic blends.

Section S2. The machinery of the novel casting method.

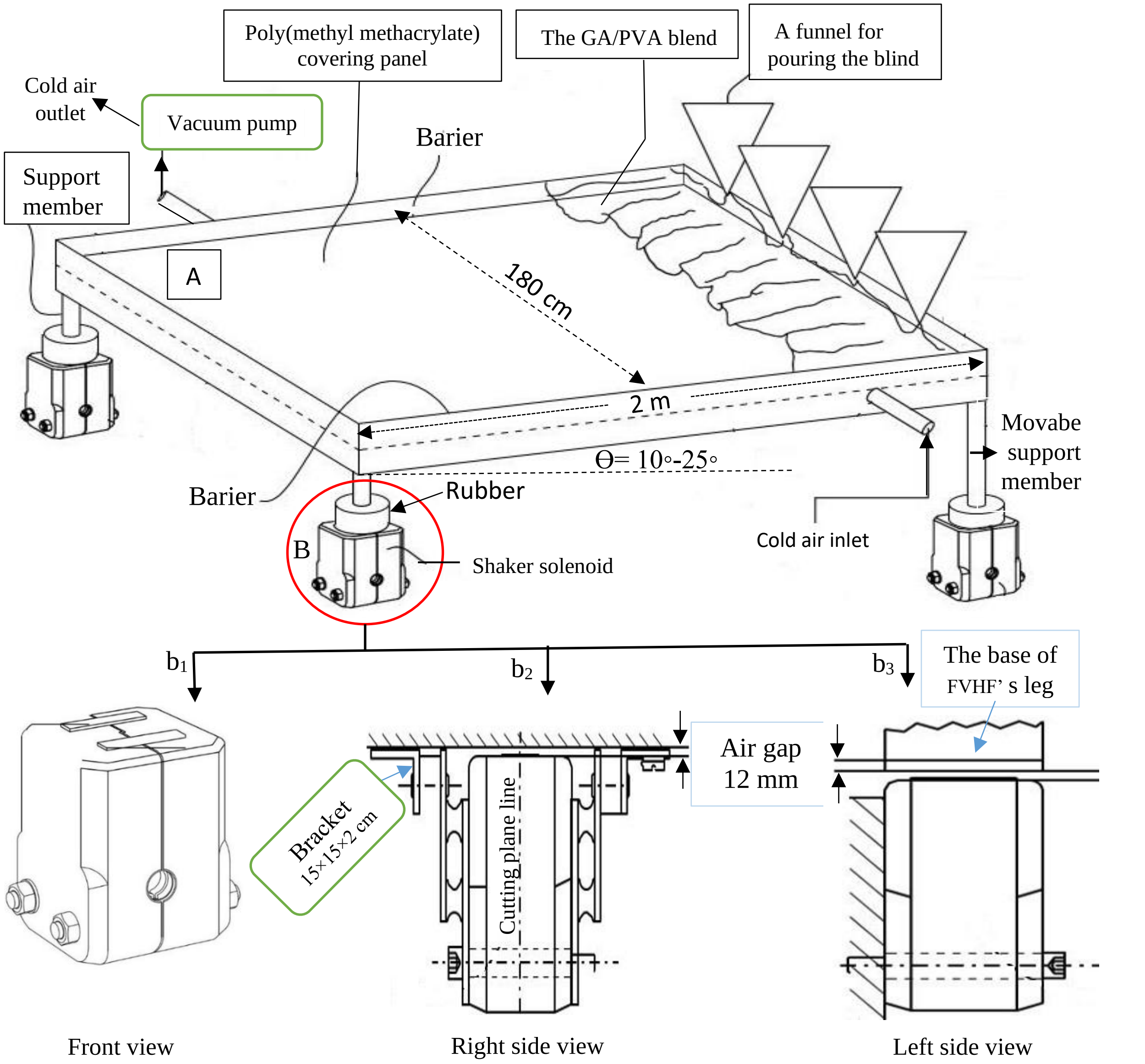

Figure S2. The casting process of the polymeric blends: A) the novel free vibrated-horizontal flow (FVHF) apparatus, and B) the magnetic vibrating system (shaker solenoid): b_1) front view; b_2) right side view showing the operational air gap (12 mm, thickness); b_3) left side view [57].

Section S3. Chemophysical features of the polymers used in the bioplastic fabrication.

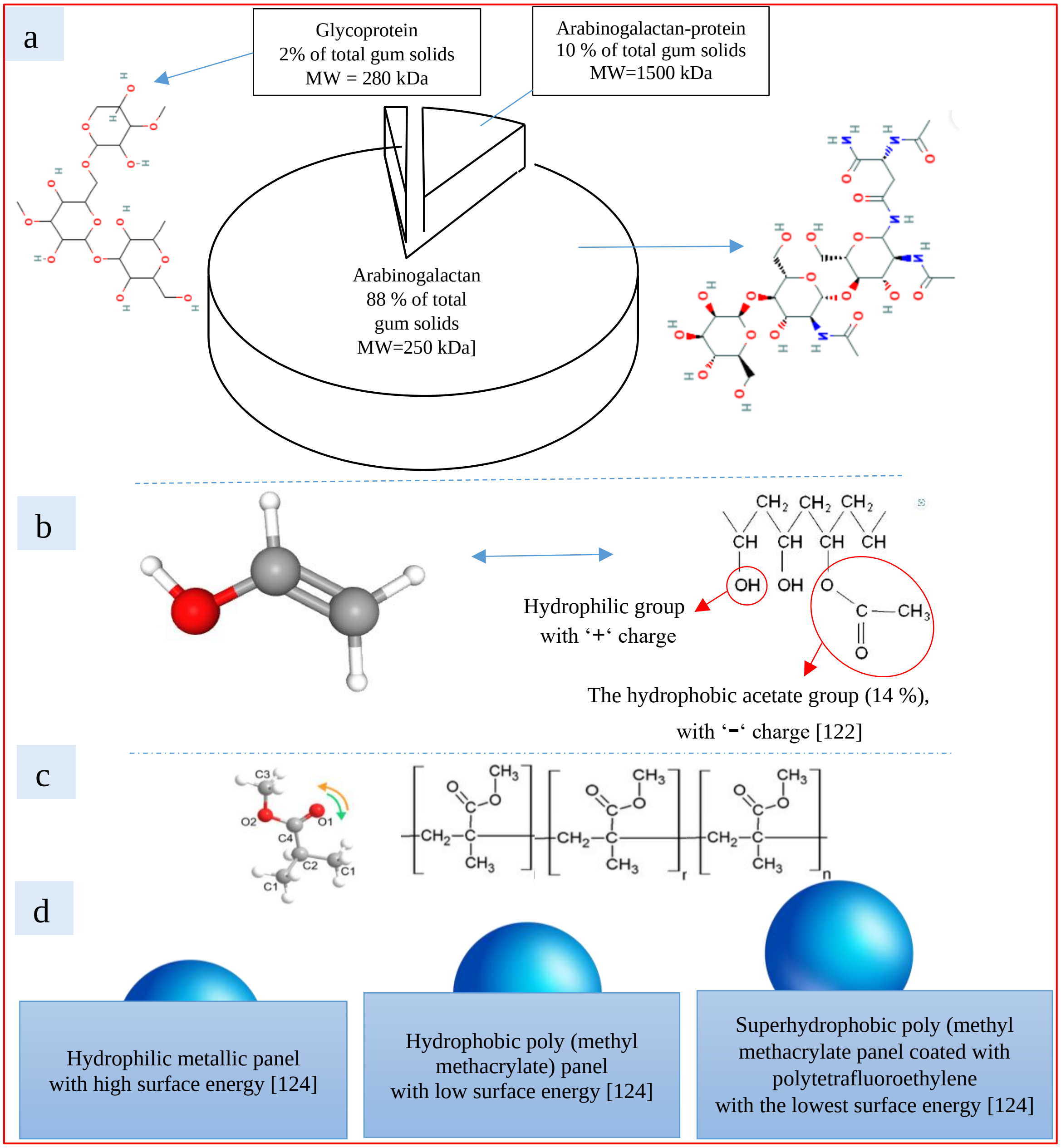

Figure S3. Chemical and physical features of the polymers used in manufacturing the NDB- membranes: a) gum Arabic (GA) precursor, b) polyvinyl alcohol (PVA) precursor, c) [poly(methyl methacrylate), PMMA] substrate [57.Hindi,Mona **2023**,93.Hindi et al 2017a, 114.Anonymous[e] 2023-115.Anonymous[f] 2023-116.Anonymous[g] 2023-117.Anonymous[h] 2023-118.Schmidt 1994, 122.Randall 1989].

Section S4. Wettability of different types of plastics.

Table S1. Surface energy and contact angle of the most important industrial polymers [124.Elsabee 2012].

Polymer abbreviation	Polymer name	Surface energy dynes/cm	Contact angle degree^{-1}
PVA	Polyvinyl alcohol	37	10
Pes	Polyester	41	70
PS	Polystyrene	34	72
SAN	Styrene acrylonitrile	40	74
PC	Polycarbonate	46	75
PPO	Polyphenytene oxide	47	75
PET	Polyethylene terephthalate	42	76
PVDF	Polyvinytidene fluoride	25	80
ABS	Acrylonitrile butadiene styrene	35	82
PMMA	Poly (methyl methacrylate)	41	82
PI	Polyimide	40	83
PU	Polyurethane	38	85
PPS	Polyphenylene sulfide	38	87
PE	Polyethylene fluoride	30	88
PBT	Polybutylene teraphthalate	32	88
PP	Polypropylene	30	88
PVCp	Polyvinyl chloride, plasticized	53	89
PES	Polyethersulfone	46	90
PVCr	Polyvinyl chloride, rigid	39	90
FEP	Fluorinated ethylene propylene	20	98
PDMS	Polydimethyl siloxane (silicone elastomer)	23	98
PTFE	Polytetrafluoroethytene	19	120
NR	Natural rubber	24	n.d.

n.d.: not defined.

Section S5. Thermogravimetric analysis (TGA) nanodehydrated-bioplastic (NDM) membranes

Table S2. Calculating [1-3] means [4,5] of mass loss (M_L) of the NDB membranes blended from gum Arabic (GA) and polyvinyl alcohol (PVA) in the six ratios and different temperature zones (T-zones).

T-zones °C	Mass loss (%)					
	GA/PVA ratio					
	1:0	1:0.25	1:0.5	1:0.75	1:1	0:1
50°-100°	**I_W**[6]. 15.64<br>**F_W**[7]. 13.5<br>**M_L**. 13.68	**I_W**. 9.62<br>**F_W**. 9.18<br>**M_L**. 4.57	**I_W**. 16.5<br>**F_W**. 15.7<br>**M_L**. 4.85	**I_W**. 15.95<br>**F_W**. 15.15<br>**M_L**. 5.02	**I_W**. 12.24<br>**F_W**. 11.6<br>**M_L**.5.23	**I_W**. 12.99<br>**F_W**. 12.25<br>**M_L**. 5.69
100°-150°	**I_W**. 13.5<br>**F_W**. 12<br>**M_L**. 11.11	**I_W**. 9.18<br>**F_W**. 8.6<br>**M_L**. 6.32	**I_W**. 15.7<br>**F_W**. 14.7<br>**M_L**. 6.37	**I_W**. 15.15<br>**F_W**. 14.1<br>**M_L**. 6.93	**I_W**. 11.6<br>**F_W**. 10.85<br>**M_L**. 6.47	**I_W**. 12.25<br>**F_W**. 11.15<br>**M_L**. 8.98
150-200°	**I_W**. 12<br>**F_W**. 11.5<br>**M_L**. 4.18	**I_W**. 8.6<br>**F_W**. 8.18<br>**M_L**. 4.88	**I_W**. 14.7<br>**F_W**. 13.75<br>**M_L**. 6.46	**I_W**. 14.1<br>**F_W**. 13.5<br>**M_L**. 4.26	**I_W**. 10.85<br>**F_W**. 10.5<br>**M_L**. 3.23	**I_W**. 11.15<br>**F_W**. 10.25<br>**M_L**. 8.07
200°-250°	**I_W**. 11.5<br>**F_W**. 10.7<br>**M_L**. 6.96	**I_W**. 8.18<br>**F_W**..7.64<br>**M_L**. 8.18	**I_W**. 13.75<br>**F_W**. 12.9<br>**M_L**. 6.18	**I_W**. 13.5<br>**F_W**. 13<br>**M_L**. 3.7	**I_W**. 10.5<br>**F_W**. 9.9<br>**M_L**. 5.71	**I_W**. 10.25<br>**F_W**. 9.85<br>**M_L**.3.9
250°-300°	**I_W**. 10.7<br>**F_W**. 9.9<br>**M_L**. 7.48	**I_W**. 7.64<br>**F_W**. 6.5<br>**M_L**.14.9	**I_W**. 12.9<br>**F_W**. 11.3<br>**M_L**. 7.36	**I_W**. 13<br>**F_W**. 11.6<br>**M_L**. 10.77	**I_W**. 9.9<br>**F_W**. 8.75<br>**M_L**. 11.62	**I_W**. 9.85<br>**F_W**. 8.85<br>**M_L**. 10.15
300°-350°	**I_W**. 9.9<br>**F_W**. 7.7<br>**M_L**. 22.2	**I_W**. 6.5<br>**F_W**. 5.68<br>**M_L**.12.62	**I_W**. 11.3<br>**F_W**. 9.5<br>**M_L**. 15.93	**I_W**. 11.6<br>**F_W**. 9<br>**M_L**. 22.41	**I_W**. 8.75<br>**F_W**. 6.25<br>**ML**. 28.57	**I_W**. 8.85<br>**F_W**. 7.25<br>**M_L**. 18.08
350°-400°	**I_W**. 7.7<br>**F_W**. 6.3<br>**M_L**. 18.2	**I_W**. 5.68<br>**F_W**.4.48<br>**M_L**.21.13	**I_W**. 9.5<br>**F_W**. 7<br>**M_L**. 26.32	**I_W**. 9<br>**F_W**. 6<br>**M_L**. 33.3	**I_W**. 6.25<br>**F_W**. 4.1<br>**M_L**. 34.4	**I_W**. 7.25<br>**F_W**. 4.05<br>**M_L**. 44.14
400°-450°	**I_W**. 6.3<br>**F_W**. 4.5<br>**M_L**. 28.6	**I_W**. 4.48<br>**F_W**. 3.24<br>**M_L**.27.68	**I_W**. 7<br>**F_W**. 4.1 mg<br>**M_L**. 41.43	**I_W**. 6<br>**F_W**. 3.65<br>**M_L**. 39.17	**I_W**. 4.1<br>**F_W**. 2.2<br>**M_L**. 46.34	**I_W**. 4.05<br>**F_W**. 0.85<br>**M_L**. 79.01
450°-500°	**I_W**. 4.5<br>**F_W**. 3.8<br>**M_L**. 15.6	**I_W**. 3.24<br>**F_W**. 2.64<br>**M_L**.18.52	**I_W**. 4.1<br>**F_W**. 3.2<br>**M_L**. 21.95	**I_W**. 3.65<br>**F_W**. 2.75<br>**M_L**. 24.66	**I_W**. 2.2<br>**F_W**. 1.2<br>**M_L**. 45.5	**I_W**. 0.85<br>**F_W**. 0.35<br>**M_L**. 58.82

[1] Using the following formula: M_L, %= [{(I_W-F_W)/I_W}×100], where

[2] Initial starting weight per mg of the GABM sample.

[3] Final starting weight per mg of the GABM sample.

[4] Each value is an average of 3 samples.

[5] Based on original oven-dry weight.

Section S6. Estimation of pore diameter of the bioplastic membranes.

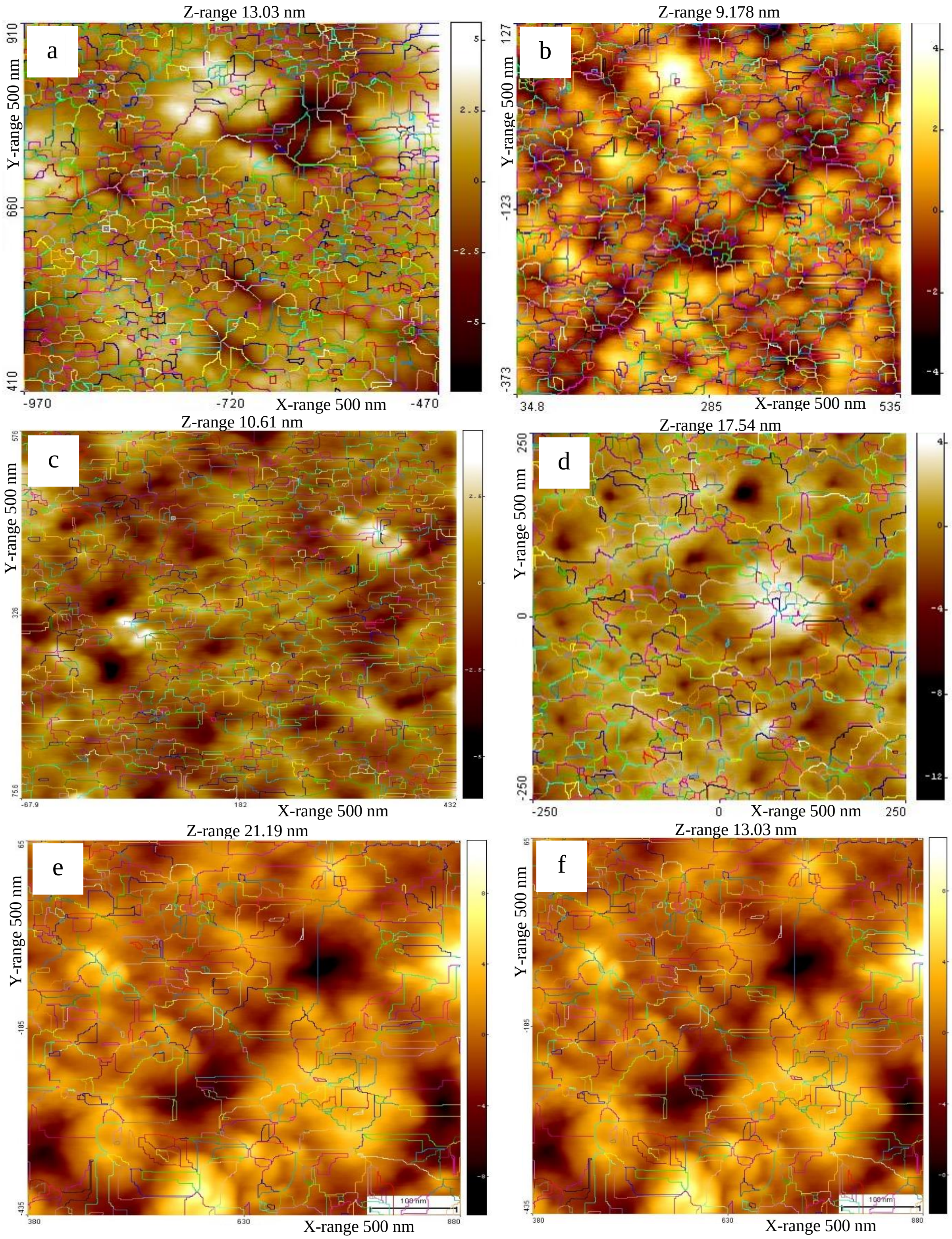

Figure S4. Visualization analysis of pore diameter (PO) of the six nanodehydrated-bioplastic (NDB) membranes (NBMs): a) GA (100%), b) GA/PVA=1:0.25, c) GA/PVA=1:0.5, d), e) GA/PVA=1:0.75, and f) GA/PVA=1:1, and (PVA=100%) based on AFM-image analysis.
